# Paracetamol Responses Depend on Temperature, Life Stage, and Genetic Background in *Drosophila melanogaster* Across Generations

**DOI:** 10.3390/medicines13030024

**Published:** 2026-07-30

**Authors:** Birk N. R. G. Hundebøl, Jesper G. Sørensen

**Affiliations:** Department of Biology, Aarhus University, 8000 Aarhus C, Denmark

**Keywords:** drug exposure, acetaminophen, toxicological effects, model organism, epigenetic inheritance, generational effects, genetic variation, *Drosophila* Genetic Reference Panel

## Abstract

Background: A key obstacle in toxicological research is investigating long-lasting or inherited effects and addressing the potential interaction between exposure and genetic background of the test subject. Such effects can be effectively investigated in short-lived and genetically managed model organisms. Methods: To illustrate the biological complexity of the outcomes of toxicological experiments, we investigated the influence of life stages, sex, and temperatures in a generational study using paracetamol exposure as an example of drug toxicology. To also include genetic background, we used five highly inbred *Drosophila melanogaster* lines from the *Drosophila* Genetic Reference Panel (DGRP). First, we assessed acute survival to paracetamol exposure at 19 °C and 25 °C, revealing clear genotype-specific dose–response curves and increased toxicity at lower temperature. We then conducted a generational experiment across three generations, in which flies were exposed during either the larval or adult life stage, and measured starvation tolerance and spontaneous activity to capture direct, intergenerational, and transgenerational effects. Results: Results show that life stage, sex, and genotype each shaped the phenotypic outcome of paracetamol exposure. Adult exposure consistently decreased activity and increased starvation resistance, whereas juvenile exposure produced more variable and sex-dependent responses. Genotype further modulated these patterns, including markedly elevated lethality in one line at high paracetamol concentrations. Conclusions: These findings demonstrate that exposure timing and biological context critically influence toxicological outcomes, underscoring the need for methodological awareness when interpreting generational studies in model organisms and beyond.

## 1. Introduction

Exposure to naturally occurring or artificially introduced stressors can influence the expression of life history traits in the offspring generation(s) of the exposed organism. These responses are known as generational effects and can both function as adaptive responses to increase resilience against the associated stressor, or can result in adverse effects and decreased fitness in subsequent generations [1,2,3,4]. Factors that have been shown to cause generational effects include both naturally occurring starvation [5,6], heavy metal exposure [7,8], and temperature changes [9,10] or contact with man-made molecules, such as pesticides [11,12], specific plastic compounds [11,12,13], or certain drugs [14,15,16,17]. Generational effects are divided into intergenerational effects, where the offspring experience direct exposure within the parent as either reproductive cells or reproductive precursors, or transgenerational effects, where only the individual’s ancestor has had contact with the stressor in question [3].

Depending on the timing of germline differentiation within a species, the distinction between inter- and trans-generational effects can become obscured in generational experiments. However, knowledge about the reproductive mechanisms for a species allows for a clear separation. In *Drosophila*, germline differentiation occurs in early oogenesis [18]. This means that using eggs produced by a female during exposure creates intergenerational inheritance in the F2 offspring, while using eggs produced post-exposure leads to transgenerational inheritance, as the F2-offspring germline has not experienced the stressor directly.

As studying generational effects requires multiple generations, investigations are both logistically and ethically complicated, and organisms with short generation times are used as essential models for longer-lived species like humans. However, the use of model organisms can have their own challenges, such as when extrapolating from the model to the target organism [19,20], and experimental procedures should be chosen carefully to best ease the gap. For example, it is important to take differences in life history and physiology into account when selecting methods for exposing the model species, even when that species contains the relevant genetic or functional homology of the target species [19,20].

The common drug, paracetamol, is used here with the model *D. melanogaster* to illuminate complexities of design and interpretation of generational experiments. Paracetamol is an analgesic and antipyretic drug, which in mammals influences prostaglandin synthesis within the central nervous system (CNS) [21,22,23]. There is further evidence that paracetamol affects the cannabinoid receptors through AM404. This metabolite inhibits anandamide uptake and functions as an agonist for TRPV1, both of which induce effects on the CNS through activation of the cannabinoid 1 (CB1) receptor [22,23]. CB1, serotonergic pathways, and TRPV1 presumably relate primarily to paracetamol’s analgesic effect [22,23], but the cannabinoid system overall also encourages feeding behavior, liver glucose production, and adipose storage, as well as some influence on pain perception [24,25]. Paracetamol may further influence mitochondrial metabolism and increase radical oxygen species (ROS) through the production of the metabolite NAPQI [22,26,27,28]. While the exact mode of action in insects has yet to be fully described, it does show toxicity [21,29] and has generational phenotypic effects [30]. Of the effects of paracetamol exposure within insects, we chose to study potential neurological and metabolic responses in this investigation.

A previous investigation suggested that generational effects have a genetic component, likely due to different tolerances to paracetamol exposure [30]. The extent to which a drug or stressor affects an organism can have a strong basis in its genetic background [31,32,33], and in humans paracetamol efficacy and toxicity varies depending on patient genetics as well as their lifestyle [34,35,36]. If this also influences the generational patterns of drug exposure, it could result in very complex interactions across generations. To account for genetic variation in this investigation, we used five nearly homozygous inbred lines from the *Drosophila* Genetic Reference Panel (DGRP) [37].

As a holometabolous insect, *D. melanogaster* has multiple and vastly different life stages, which have unique behavior and physiology [38]. One change between life stages is the near total demethylation of DNA within adult flies, which is thought to happen during the pupal stage [39,40,41], potentially resetting or decreasing epigenetic signals of methylation induced during the larval stage. Early life stages are particularly sensitive to environmental changes [42,43], which may alter growth, reproductive success, and gamete development [42,43]. In addition, as ectothermic organisms the metabolism of insects is also very sensitive to temperature changes [44,45]. This causes complex interactions involving temperature in ecotoxicological studies, as temperature both directly and indirectly affects toxicity. Such indirect effects might arise through temperature dependency of larval developmental times [46,47,48], which in turn affects length of exposure.

Here, we explore how biological parameters influence the outcomes of a generational toxicological investigation using the model *D. melanogaster*. The potential parameters investigated are temperature, genetic background, and life stage, and they are assessed for direct, intergenerational, and transgenerational responses using paracetamol as an example drug. To select the appropriate life stage and temperature-specific exposure concentrations, we create paracetamol dose–response relationships for egg-to-adult survival at two temperatures. Noting that toxicological dynamics among ages and life stages can vastly differ, we make no absolute comparisons or assumptions regarding internal concentrations but base our study on relative responses to exposure. We evaluate paracetamol effects on spontaneous locomotor activity, as a proxy for neurological effects, and starvation tolerance, as a proxy for changes to metabolism, across three generations. The method of spontaneous locomotor activity is prioritized as behavioral assessment over geotaxis or startle-based assays because it provides a low-stress and easily reproducible evaluation, which minimizes acute stress before the same flies undergo the subsequent starvation assay. This reduces the risk that assay-induced arousal or energy expenditure confounds starvation tolerance.

We hypothesize that the negative effect of paracetamol exposure on egg-to-adult viability in *D. melanogaster* is exacerbated at low temperatures (as exposure time increases) and as paracetamol doses increase. We hypothesize that paracetamol sensitivity is higher when developing stages are exposed as compared to adults. Though directionality of responses of distinct genetic lines cannot be easily predicted, we hypothesize that paracetamol has genotype-specific effects on spontaneous locomotor activity and starvation tolerance in directly exposed (F0) flies and as generational effects across the F1 and F2 offspring generations.

## 2. Materials and Methods

### 2.1. DGRP Lines

We selected five random lines of *D. melanogaster* from the *Drosophila* Genetic Reference Panel (obtained from Bloomington *Drosophila* Stock Centre; NIH P400D018537). The identities of the relevant lines, from now on called Lines 1–5, can be seen in Table S1. At the time the investigation started, the flies had been maintained at the laboratory for approximately two years at 19 °C with a 12:12 light:dark cycle on a standard *Drosophila* oatmeal–sugar–yeast–agar medium [49]. Flies used for the generational experiment were moved to 25 °C with a 12:12 light:dark cycle and acclimated for two generations before initiating the experiments.

### 2.2. Temperature Experiment

A dose–response curve was made to investigate the effect of temperature on paracetamol tolerance in *D. melanogaster.* We assayed 7 different concentrations at 19 and 25 °C using the same methods as described in our previous investigation [30]. Vials of fly food were made using approximately ~2 g of instant Carolina fly food (Formula 4-24^®^Instant *Drosophila* Medium, Carolina Biological Supply, Burlington, NC, USA) with water containing concentrations of either 0, 5, 10, 20, 35, 50, or 75 mM of paracetamol (4′-hydroxyacetanalide, Merck, Darmstadt, Germany, CAS-No 103-90-2). Fly exposure was achieved by adding exactly 50 eggs per vial, with 3 vials per line for each dose by temperature combination. The synchronized eggs (10 ± 8 h old) were collected from food bottles where batches of 40–100 flies were left to oviposit overnight. The number of eclosing adults was counted for each vial to create a survival dose–response curve.

### 2.3. Generational Experiment

The generational experiment investigated generational effects on spontaneous locomotor activity and starvation tolerance for five different treatment groups across three consecutive generations, with the F0 generation being the exposed individuals and the following two generations of non-exposed offspring (F1 and F2). Two groups consisted of juveniles subjected to 20 (J20) or 40 mM (J40) of paracetamol within their food throughout larval development. Similarly, two groups represented adult exposure to 40 (A40) or 80 mM (A80) of paracetamol in their food for five days. A final control group (C) was living on unexposed Carolina fly food in similar time increments as the other groups. Juvenile doses were determined using results from the temperature experiment, while adult exposure was increased to account for a shorter exposure time (larval developmental time = approximately 14 days, adult exposure = 5 days). Doses were aimed at being sub-lethal and thereby inducing as strong responses as possible without substantial mortality and thereby selection. As exposure dynamics and accumulation are markedly different between developing and adult life stages, we assume neither comparability of absolute exposure concentrations nor do we directly compare absolute doses or responses between experiments performed on developing and adult life stages. However, the design allowed the identification of a general pattern of directionality and proportionality.

For each DGRP line, ten vials of Carolina fly food were prepared, as described above. Six vials were unexposed to prepare for treatments C, A40, and A80, while the remaining contained paracetamol (4′-hydroxyacetanalide, Merck, Darmstadt, Germany, CAS-No 103-90-2) dissolved in water at appropriate concentrations to ensure treatments J20 and J40 had two vials each. F0 was then established by adding 50 ± 5 eggs (collected as described above) to each vial to ensure development in density-controlled conditions. Approximately three days after adult eclosion, all flies were moved to new vials of Carolina fly food for five days, and flies from four out of the six unexposed vials were put on food containing appropriate paracetamol concentrations for treatments A40 and A80 to have two vials each. Once initial paracetamol exposure was established, flies were kept on standard *Drosophila* (oatmeal–sugar–yeast–agar) medium. F1 and F2 generations were established by mixing offspring from different vials and allowing egg laying by excess adult flies from each treatment over a day. As we use eggs collected from adult females >3-day post-exposure, we assume that the detected responses of F2 offspring are transgenerational.

#### 2.3.1. Spontaneous Locomotor Activity

Investigation of spontaneous locomotor activity in F0 occurred on day ten—three days after flies were moved away from the Carolina fly food to allow recovery from paracetamol exposure within A40 and A80 flies. Male flies were assayed first so females could oviposit eggs to establish future generations. This meant males flies were 9–13 days at activity assay, while females were 11–15 days at activity assay.

Spontaneous locomotor activity was investigated using video tracking, as described in a previous investigation [30]. Flies were put into arenas to be recorded in illuminated heating cabinets at 25 °C. Noldus Ethovision video tracker version 16 [50,51] was used to track spontaneous locomotion in the videos, noting the distance moved (cm) at a resolution of 2 data points per second. Not all samples were perfectly tracked, leading to missing information (NA in the dataset), and any flies with above 30% missed data points were excluded as quality control. Data were averaged across bins corresponding to each minute of the trial, creating multiple individual points of average speed per minute for each fly for the final analysis. Data from the first 10 min after initiation of the trail were excluded to allow the flies to settle down after handling.

#### 2.3.2. Starvation Assay

Flies were provided with supplementary yeast on the day prior to the starvation investigation to give an even baseline between flies, and the investigation was initiated on the day of the activity investigation. Once the activity arena was full excess flies were put in a vial without fly food until the prior investigation was finished. Flies were then transferred to petri dishes with plates made from 2% agar as their water supply and kept at approximately 25 °C. A camera was placed above the petri dishes, and a picture was taken every hour until all flies had stopped moving. The pictures were used to assess whether flies had changed position between pictures, and time-to-death (TTD) was assigned at the earliest hour each fly had reached its final position and lost its ability to stand upright.

### 2.4. Statistics

All statistics were performed in R version 4.5.2 [52], and model diagnostics (residual checks, model fits, dispersion, collinearity, and model comparison) were performed as appropriate for the model fit. Visualizations were done using the ggplot2 package [53] unless otherwise stated.

#### 2.4.1. Temperature Experiment

To investigate the dose–response curve, a binomial model was created using the lme4 package [54] for modeling, the DHARMa package [55] for model verification, and lmerTest [56] and car [57] to obtain tests for the fixed effects. Survival was used as the response variable, dose, temperature (Temp), and line were used as fixed effects, while vial ID was included as a random effect to stabilize estimation under near complete separation, as mortality surrounded 100% at doses 50 and 75 at 19 °C.

Logistic, quadratic, and cubic dose–response forms were tested and compared for best fit to accommodate a potential hormetic effect using the glmer function. Relevant interactions were identified by comparing AIC, BIC, logLik, and diagnostic fits with preference for simpler models, and dose was scaled to stabilize the fit. Parametric bootstrapping was then used to create standard errors, a bias estimate, and confidence intervals (CIs) for the final model using bootMer with 200 replicates. Final model fit ended with a quadratic fit for dose with the following interactions to avoid inflated coefficients and overfitting:

$$Survival\;\sim \;scaled\left(Dose\right)\cdot \;Temp\;\cdot \;Line+scaled{\left(Dose\right)}^{2}\;\cdot \;Temp\;+\;scaled{\left(Dose\right)}^{2}\cdot \;Line+(1\;|\;Vial\text{\_}ID).$$

A total of 10,450 eggs were divided between 209 vials for the dose–response curve. Final results were presented using a type II χ^2^ test in an analysis of deviance table.

#### 2.4.2. Spontaneous Locomotor Activity

To investigate spontaneous locomotor activity, a hurdle approach was adopted to accommodate the cluster around zero, likely attributed to sensing noise rather than actual movement. All model diagnostics were assessed using the DHARMa package [55], results were procured with the car package [57], and post hoc analyses were performed using the multcomp [58] and emmeans packages [59]. Each sex was modeled separately to accommodate differences in age and potential other confounders. A density plot was created for the log-transformed distance (Dist) to determine the threshold for movement. A binomial model with a logistic regression link was created for each full dataset to analyze active and passive flies using the glmmTMB package [60,61].

Fixed effects included treatment (Treat), generation (Gen), and line, with a random intercept for individual fly ID included to account for repeated measures across flies. Model structure was determined through AIC comparison with a preference for simpler models to achieve a stable and accurate fit. The final model structure for both sexes was as follows:

Activity ~ Treat·Gen+Line·Gen+Treat·Line+(1 | ID).

According to the density blot, the threshold for movement sat at 0.018 cm moved. This produced a split of 18,850 active and 22,662 passive minutes for females across 1185 flies, while males had 32,876 active and 12,333 passive minutes across 1275 flies within the assay time.

A separate set of models was created to investigate the continuous movement patterns of the active flies using the glmmTMB package [60,61]. Based on diagnostics, the female data best fit a beta regression with a logit link, with a linear model best representing the male activity data. Using the same parameters and model selection as within the binomial model, the final model structure for both models is as follows:

Distance ~ Treat·Gen+Line·Gen+Treat·Line+(1 | ID).

ID contributed markedly to variance within all models. Post hoc comparisons were performed for each model to extract the differences between treatments, using Holm–Bonferroni p-adjustment to mitigate alpha-inflation. A first comparison was made within each generation by averaging effect across the lines. A second comparison was then made to compare treatment effects within each line for each generation. Compact letter displays (CLDs) were created to ease interpretation.

#### 2.4.3. Starvation Assay

To investigate starvation tolerance, multiple survival models were tested using the survival package [62,63], with visual inspection of observed versus modeled survival curves to confirm fit. Post hoc analyses were further performed using the multcomp [58] and emmeans packages [59]. As with spontaneous locomotor activity, each sex was modeled separately to accommodate differences in age and potential other confounders. Fixed effects included treatment (Treat), generation (Gen), and line, with clustering for individual experimental unit ID included to account for differences in microenvironment.

Accelerated failure time models were selected for the final analysis. The female model settled on a Weibull distribution with 1875 individual flies observed across 128 petri dishes, while the male distribution was fitted to a lognormal setup and included 1758 flies across 138 dishes. Flies dying within 10 h were censored as starvation-independent mortality, as this might be associated with handling or bad condition rather than starvation. Female flies lasting more than 80 h were further censored as well to minimize the influence of a long tail, but the male data showed no such behavior. For both models, the final model structure is as follows:

$$Time\;til\;Death_{Hours}\;\sim \;Treat\cdot Gen+Line\cdot Gen+Treat\cdot Line+cluster(Dish_{ID}).$$

Petri dish ID contributed markedly to variance within both models, showing the necessity of the clustering. Post hoc comparisons were performed for each model to extract the differences between treatments on the generation level by averaging effect across the lines, and Holm–Bonferroni p-adjustment was used to mitigate alpha-inflation. Comparisons were also made on the levels of generation and line, and CLDs were created to ease interpretation.

## 3. Results

### 3.1. Temperature Experiment

Increasing concentrations of paracetamol significantly increased fly mortality, and low temperatures also elevated mortality significantly. A significant effect also validated the presence of the quadratic dose term, showing a statistically significant hormetic effect of paracetamol on fly survival. Lines further responded differently from each other, and they also showed varying degrees of paracetamol and temperature tolerance, confirming that genetic background modulated sensitivity to both paracetamol and temperature. The significant interactions between the quadratic dose term and temperatures showed that the early protective effect on paracetamol depended on both temperature and genotype. The details of the statistical analysis can be seen in Table 1.

**Figure 1:**
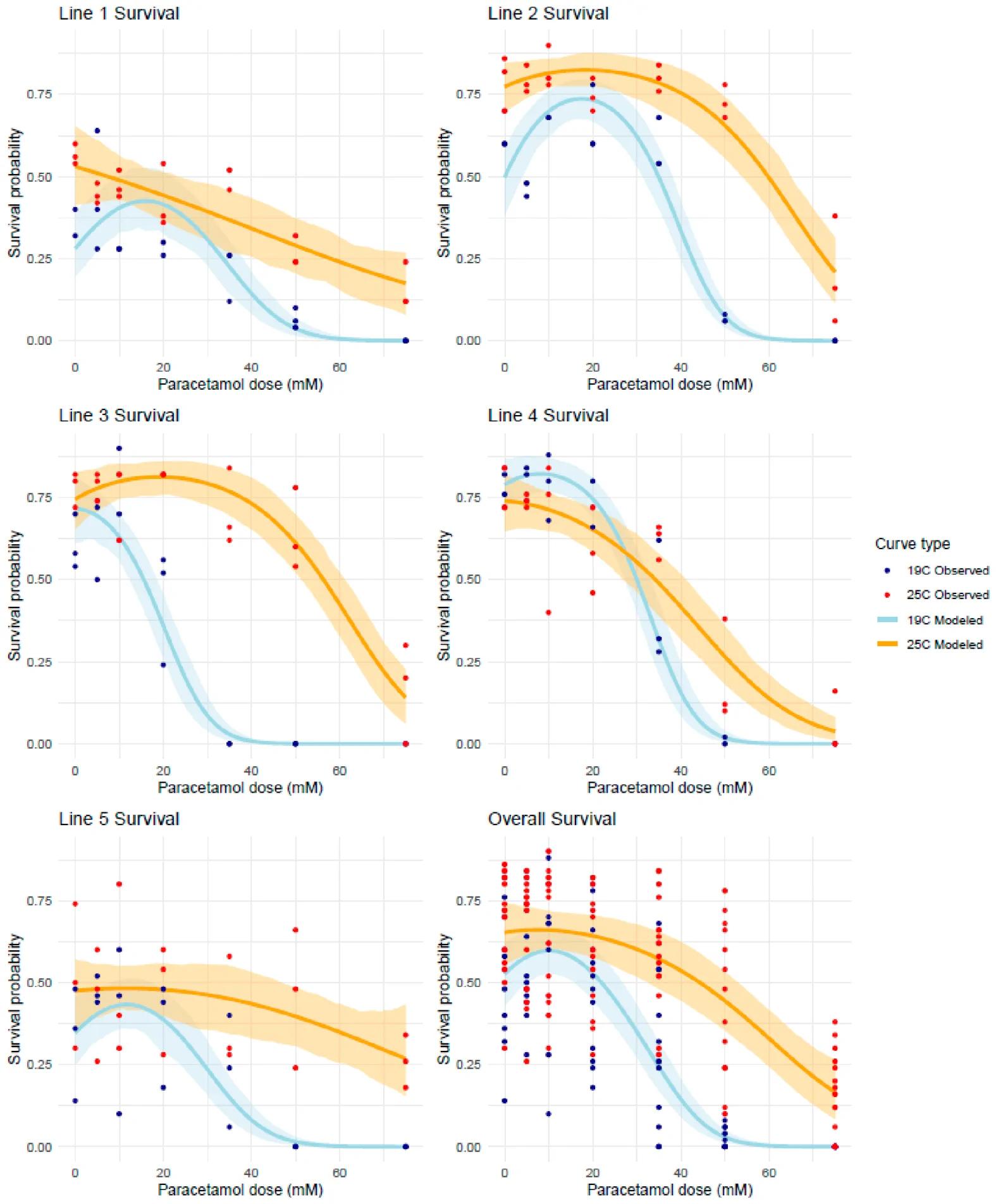
Overview of the dose–response curves for the temperature experiment, showing the predicted curves with associated 95% confidence intervals as well as the observed values as dots, averaged for each vial. The lower right corner presents the overall effects averaged across lines, while each preceding graph shows per line responses.

Overall, the model confirms a complicated genotype-by-environment effect due to the presence of significant main effects and both two-way and three-way interactions, though the model was generally well behaved. The scaled model residuals were well centered, though the maximum residual reached 7.24, reflecting the high mortality at extreme doses. The random vial intercept showed only a modest effect, indicating that a majority of variance was explained by the fixed effects. The full statistical overview can be seen in Table S2, and a visual overview can be seen in Figure 1.

### 3.2. Generational Experiment

#### 3.2.1. Spontaneous Locomotor Activity

The binomial regression models for both sexes revealed complex treatment, line, and generation effects on the likelihood of flies moving, including for their interactions (see Table 2). On average, females had a 45.4% probability of moving, while for males it was elevated to 72.7%. This is reflected in the intercept, which was not significantly different from zero for females (β = 0.607, SE = 0.486, *p* = 0.21) while it was significantly positive for males (β = 1.36, SE = 0.504, *p* = 0.007). In contrast, males showed strong effects of the main treatments, while in females, only A40 had a significant effect (β = −1.547, SE = 0.736, *p* = 0.036). For females, flies were less likely to move in generation F1 (β = −1.545, SE = 0.603, *p* = 0.01) compared to F0, while both generations were near significant within males (F1: β = −1.04, SE = 0.616, *p* = 0.092; F2: β = 1.05, SE = 0.589, *p* = 0.075). There were clear interaction effects between lines and treatments as well as treatment and both generations within each sex, suggesting the presence of inter- and trans-generational effects. This, added with line effects interacting with both generation and treatments, reflects highly complex patterns.

The continuous models, a beta regression with a logit link for females and linear regression for males, also showed effects of all fixed effects and their interactions on distance moved once flies were active (see Table 2). Mean distance traveled was 0.159 for females and 0.195 for males. Of treatment effects, A40 once more significantly decreased distance compared to control females (β = −0.843, SE = 0.169, *p* < 0.001), while the only significant treatment for males was J20, which increased the average distance by 0.048 cm (SE = 0.023, *p* = 0.035). Both subsequent generations were less active than F0 within females (F1: β = −0.69, SE = 0.136, *p* < 0.001; F2: β = −0.49, SE = 0.126, *p* < 0.001), while only F2 showed change in males, increasing distance by 0.081 (SE = 0.017, *p* < 0.001). There was a strong interaction between all treatments and generation for females except A80 in F2, suggesting strong inter- and trans-generational effects. For males, some treatment-by-generation effects were present in both generations, but much less pronounced. Both showed genetic line effects, which interacted with the other terms.

Delving into line-specific responses at each generation seen in Table 3 and Table 4, J20 tended to significantly increase both the likelihood of female activity and distance moved in generations F1 and F2, with L4 as an exception to the rule. For F0, however, J20 generally matches control flies. Interestingly, J40 significantly decreased the likelihood of movement in lines L3 and L4 for F0, a trend not reflected in distance moved for the same metric. As a contrast, J40 increases both the likelihood of activity and distance for F1 and F2, representing a switch from direct exposure to generational effects. A80 increased both the likelihood of movement and distance traveled in F1 but only select lines in F2 showed increased activity and experienced no effect on distance. A40 tended to decrease distance moved overall in directly exposed flies, and L3 showed the same tendency regarding activity, though this did not translate to future generations. Opposing these overall trends, L4 responded to the A40 treatment in all generations with increased activity.

Contrasting with the female flies, males showed a decreased response to paracetamol, with especially lines L2 and L4 being less responsive in the linear model for generations F0 and F1. Further, juvenile treatments tended to have much less effect than the adult treatments. Part of this reflects a generally high activity within male flies, with most groupings staying well above 80% movement detected. For overall trends, J20 and both adult treatments increased the likelihood of activity in directly exposed flies, but no treatment altered the distance moved. For the subsequent generations, A40 decreased and A80 increased activity, but for distance that pattern only persisted in F1 for A80 and F2 for A40. This particular A40 pattern was present in all lines, but for the other generations as well as the activity model, line-specific responses were sporadic. A80 clearly showed the greatest likelihood of activity across groupings, and J40 had a similar pattern, tending to increase activity. However, only Line 5 in generation F1 reflected that pattern for the distance model, and in F2, distance instead tended to decrease. J20 tended to decrease distance in F2, though activity was increased for L1 in both F0 and F1 while remaining otherwise unresponsive.

**Figure 2:**
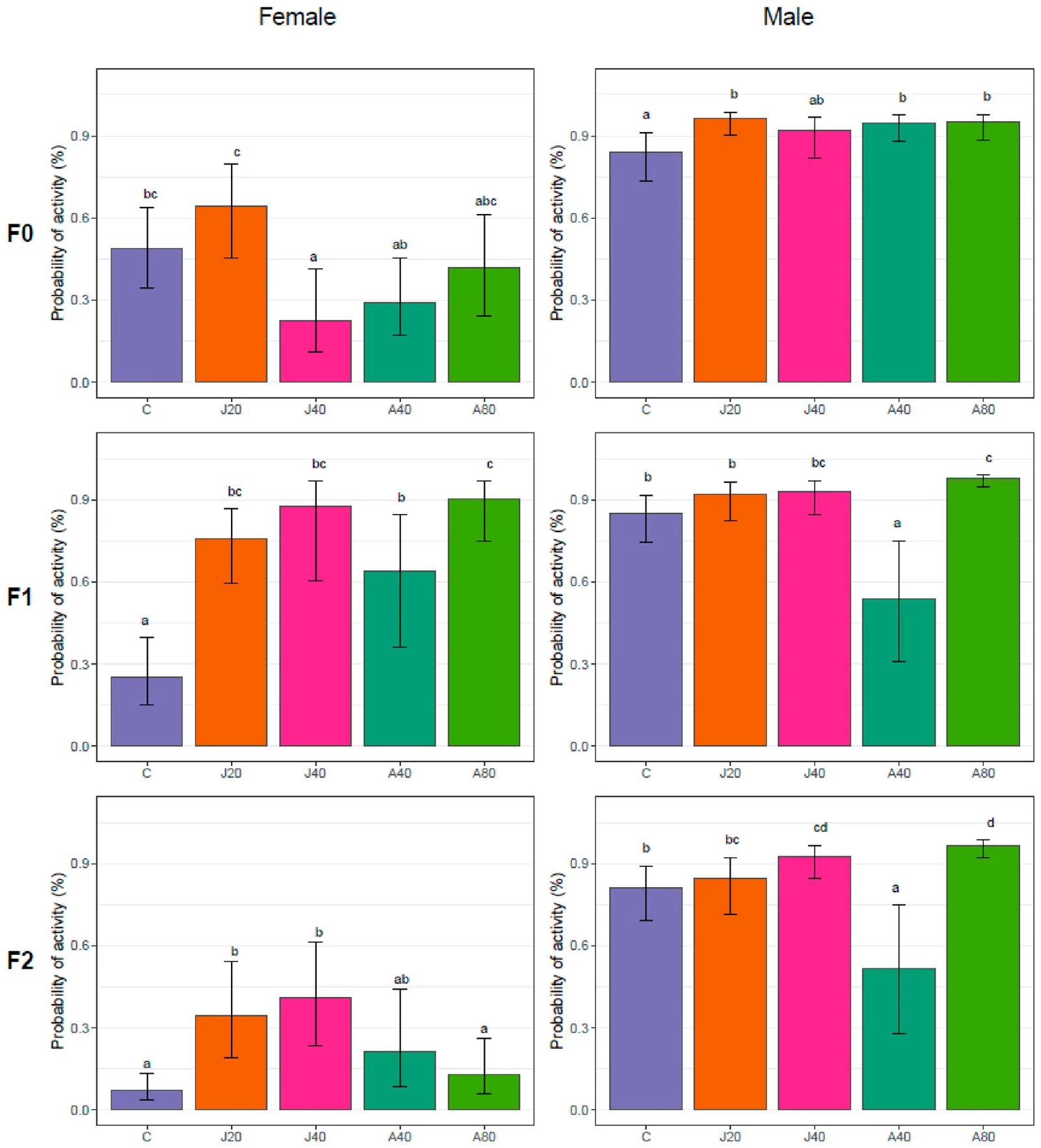
Per generation comparison between treatments for the activity model in all lines, split between the sexes. Error bars show the 95% confidence interval and different letters mark statistically significant differences among treatments within each panel. Holm’s p-adjustment was used for the post hoc comparison.

**Figure 3:**
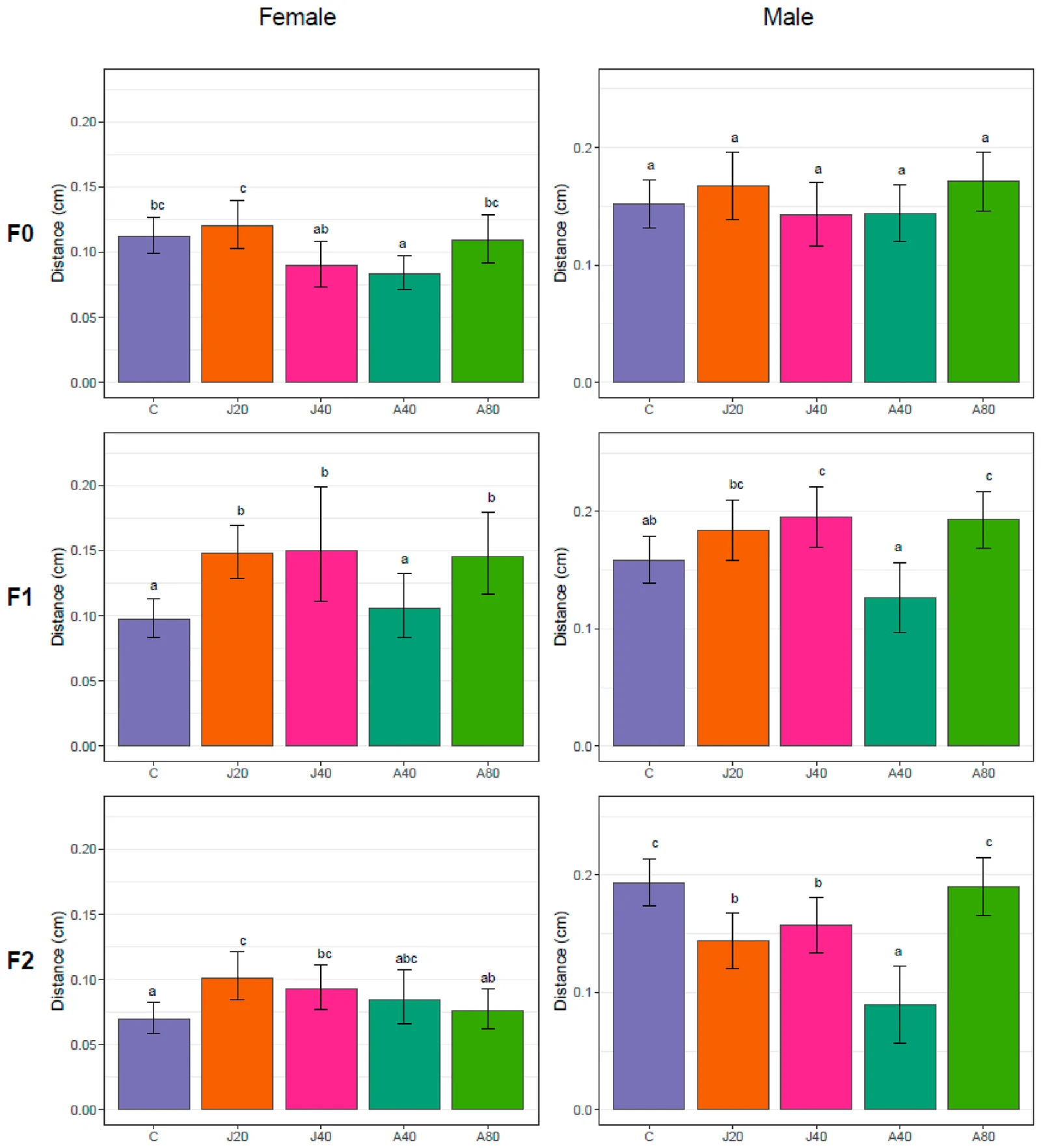
Per generation comparison between treatments for the distance model in all lines, split between the sexes. Error bars show the 95% confidence interval and different letters mark statistically significant differences among treatments within each panel. Holm’s p-adjustment was used for the post hoc comparison.

The full statistical overview can be seen in Tables S3–S14, and a visual overview can be seen in Figure 2 and Figure 3.

#### 3.2.2. Starvation Assay

The starvation models showed clear influence of treatment, line, and generation on starvation tolerance, including effects from interactions (see Table 5). While direct comparison is impossible, females showed higher starvation tolerance relative to males, reaching a median mortality after 42.3 h compared with 27.0 h for males. The scale parameter laid below 1 (σ = 0.277) for the female Weibull distribution signifies that hazard increased over time, but this inference cannot be made for the male lognormal distribution. Here, the scale parameter only determines the slope of the hazard, as lognormal hazards always increase to a peak before decreasing. Survival time decreased for females (β = −0.270, SE = 0.112, *p* = 0.022) but increased for males (β = 0.337, SE = 0.112, *p* = 0.003) in F1. Only L5 differed from the baseline L1 line in the female model (β = −0.325, SE = 0.115, *p* = 0.005), while L2 (β = −0.291, SE = 0.118, *p* = 0.027) and L4 (β = −0.556, SE = 0.130, *p* < 0.001) showed decreased survival time in males. No treatment main effect showed significance as compared to the baseline (Line L1, Generation F0), and females generally showed less sensitivity, with many interactions hovering on the cusp of significance. Only the F1:J40 interaction showing a lengthening survival effect (β = 0.276, SE = 0.118, *p* = 0.0194) and some few line interactions reached significance. Males expressed many significant interactions, with multiple treatments having interaction effects within both generations as well as multiple cases of interactions involving line. This was primarily driven by L1, which showed a long-lived phenotype.

**Figure 4:**
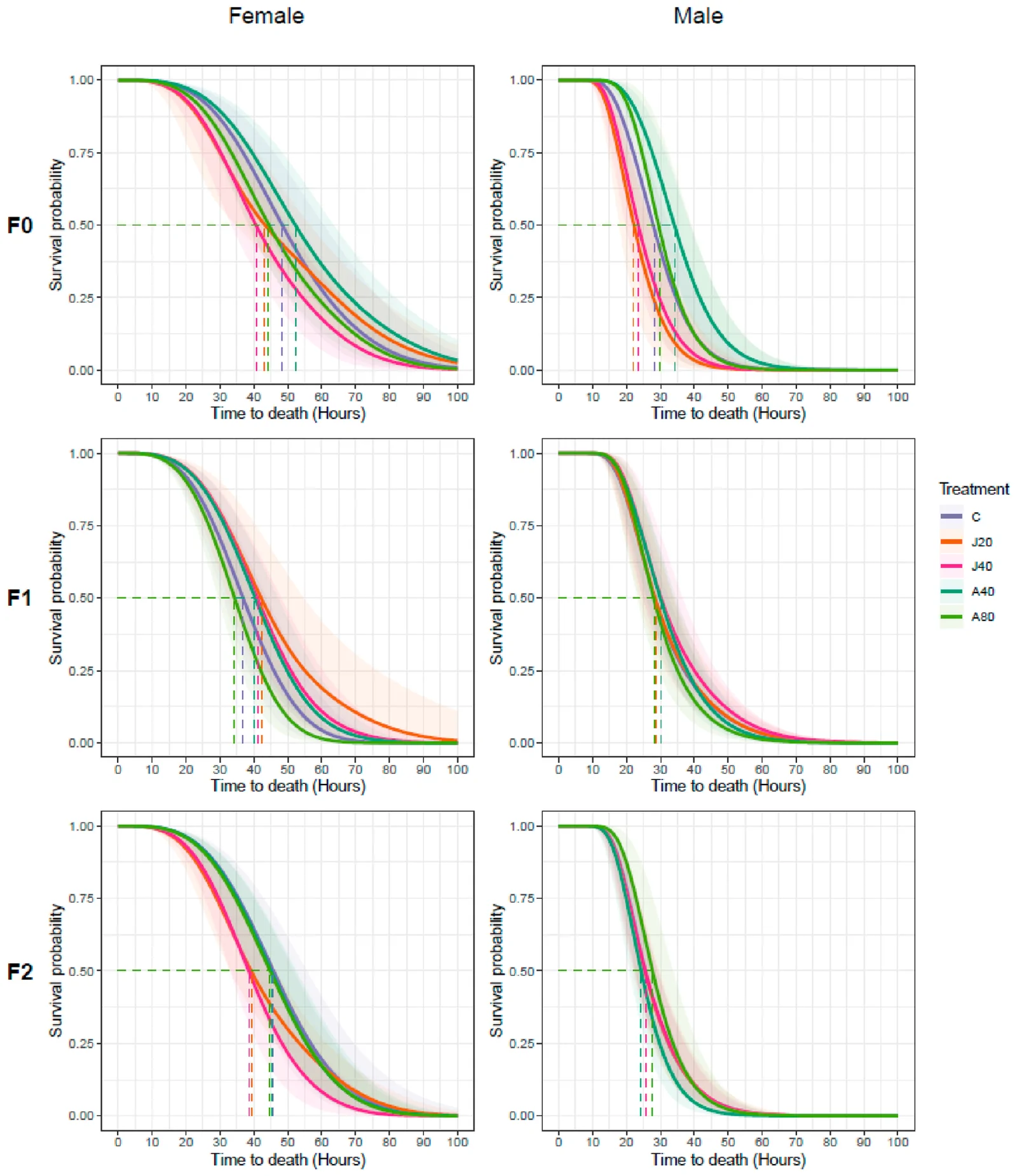
Per generation comparison between treatments for the starvation data, split between the sexes. Ribbons signify the 95% confidence intervals from the survival models.

Looking at individual treatment comparisons, both between the generations overall shown in Table 6 and for the line comparisons, there is no clear evidence of transgenerational effects. This persisted for the female model for all generations when comparing treatments against the control, but for F1 there was a significant decrease in survival time for A80 in L3 as compared to the other adult treatment, A40. Males experienced an overall increase in survival time for directly exposed flies of treatment A40 but otherwise no significant effects. The full overview can be seen in Tables S17 and S20. For male line comparisons, many of the directly exposed flies were affected by paracetamol exposure. J20 decreased the survival time for both L2 and L5, within which J40 also decreased survival. A40, on the other hand, had a positive effect on both L2 and L4. This trend for A40 continued in F1 for L2 as the only clear generational effect. Despite the general absence of generational effects on the line level, many combinations of treatment × generation × lines subgroups showed predicted median differences of 5+ h. Especially some combinations within females had a 10+ difference between the controls and some groups. The full statistical overview can be seen in Tables S15–S20, and a visual overview can be seen in Figure 4.

## 4. Discussion

This investigation revealed genotype-, sex-, and life-stage-specific responses to paracetamol, highlighting their importance in the selection of experiment design. These responses continued in future generations, following a pattern that depended on life stage of exposure. Paracetamol had a hormetic effect on egg-to-adult survival that was temperature dependent, with low temperature and higher doses resulting in increased mortality. We found a distinct pattern between male and female treatment responses in both spontaneous adult activity and adult starvation tolerance. For activity, this lasted across the two investigated offspring generations, while starvation tolerance primarily operated in directly exposed flies and their offspring. Genetic background had a strong impact on the response to paracetamol, making general conclusions regarding the effects of exposure challenging. In this study, we evaluated spontaneous locomotor activity, as a proxy for behavioral effects, and starvation tolerance, as a proxy for changes to metabolism. As the two traits showed highly variable and complex responses to paracetamol, it is obvious that other traits could have generated different results. Several other behavioral endpoints could have been chosen (e.g., startle response and geotaxis); however, as argued in the introduction, we deemed the chosen endpoints as less invasive and, therefore, preferable.

The decrease in egg-to-adult survival at a low temperature likely resulted from the longer development time and thus prolonged exposure to paracetamol [44]. Mortality to chronic exposure to paracetamol has been shown to increase over time [29]. The exact mechanism is not specified in this investigation, but could arise from increased ingestion of paracetamol, increased time within the exposed substrate, or temperature-dependent digestive responses prolonging metabolites’ presence within the organism. Awareness of the physiological effect of temperature is, therefore, necessary when using ectotherms as model species, especially if exposure to potentially toxic chemicals is involved.

Adult exposure tended to result in decreased spontaneous locomotor activity at low doses, while the high dose and exposure at the larval stage tended toward increased activity in females. These were especially pronounced as inter- and trans-generational effects. For males, low-dose juvenile treatment tended to make a transgenerational decrease to distance traveled, while the high adult dose increased the likelihood of movement and, when lines experienced a significant effect, distance traveled. Different genotypic backgrounds also showed different degrees of sensitivity toward paracetamol. Spontaneous activity responses reverted or disappeared between generations in some lines. It is very difficult to assign biological significance to the observed changes in spontaneous activity and starvation tolerance. First, the high degree of variability among lines and generations makes general conclusions difficult, potentially misleading in practice if averaged across generations, and obstructs a clear effect size estimation. Second, a small effect size does not necessarily equate with a small biological effect. Third, the starvation model produced highly different medians between treatments within subgroups, which could have real biological implications even if the setup did not pass the threshold of statistical significance in a mixed model. Thus, we refrain from making absolute conclusions about the absolute biological relevance of the observed patterns among genetic backgrounds and generations for the two sexes.

Our results on activity measurements reflect a unique sex and life-stage determined response of spontaneous activity when *D. melanogaster* individuals are exposed to paracetamol, and these are only further complicated by genotype-specific patterns to treatment. We speculate that the seemingly unpredictable responses in activity might be explained by the vast array of different metabolites produced from paracetamol, which affect several physiological processes [21,22,23]. This includes production of the reactive metabolite NAPQI, which only causes toxicity above a certain threshold [22,26,27,28]. The potential involvement of this metabolite is an obvious target of further investigation.

Exposure to paracetamol primarily tended to protect against starvation for adult exposure, increasing survival in treatments that differed from the control group. Based on knowledge from mammals, we speculate that paracetamol may affect metabolism in several ways, as both prostaglandins and cannabinoids in this taxon have a secondary impact on energy uptake and storage [24,25,64,65]. Paracetamol further influences the degree of ROS in cells [66,67], which might mitohormetically increase mitochondrial activity and efficiency [68,69,70,71]. Further, the metabolite AM404 might induce an increase in feeding behavior if an insect homolog to the cannabinoid system exists. Thus, increased food uptake in the directly exposed adult flies prior to the starvation assay could explain increased starvation tolerance. Whether this increased uptake alone exists and can translate to a transgenerational effect remains to be investigated.

When juvenile effects were significant in the starvation experiment, however, survival time tended to decrease. This may reflect the response to paracetamol exposure due to the sensitivity of environmental influences during developmental stages [42,43]. Such an effect may also be present in the activity assay, as there was an increased tendency toward hyperactivity in especially females for the juvenile treatments compared with adult exposure for both movement likelihood and traveled distance.

Clear genotype-specific responses to paracetamol exposure were identified in all parts of this study. The temperature experiment showed highly different dose–response curves depending on the line, and activity patterns across both generation and treatment regimens were line dependent. For example, approximately 45% of flies in Line 1 succumbed during exposure, which highly contrasted with the occasional single deaths observed in other lines. The increased lethality in Line 1 is not reflected as higher sensitivity in general, as flies of this line do not have a unique response within the spontaneous activity assay. Future studies could test the hypothesis that the increased sensitivity in mortality of Line 1 might be related to the dynamics of accumulating or clearing toxic paracetamol metabolites, e.g., NAPQI, as is seen within some human genotypes [34,35].

Males showed both a generally higher level of spontaneous activity and a decreased starvation tolerance. These two trends are likely correlated, as activity increases the energy consumption of an organism and thereby how quickly reserves are depleted. The fact that males are smaller than females may further extend this correlation [72], as this decreases the size of their fat storages in comparison with the opposite sex. In many cases a smaller size has been shown to increase sensitivity to toxicants [73,74], putting males at higher risk of surpassing paracetamol’s hormetic zone at lower doses than females. Should this apply, it can limit our ability to directly compare male and female responses even further than what the age difference might cause, especially when no measurement of internal dose was performed to compare the sexes.

The reason for differences between the responses in life stages is not investigated in this study. It may be specific to *Drosophila* species due to the genetic demethylation during pupation [40], or it may reflect a general feature within holometabolous insects after metamorphosis [39]. However, the basis may also be a difference in sensitivity to exposure between juvenile or adult specimens, which may translate across generations [42,43]. Finally, paracetamol ingestion may be different between adult and juvenile flies, as adult flies have shown to behaviorally avoid bitter compounds, such as paracetamol [75,76]. When investigating and extrapolating results from model organisms to humans, it may be important to consider not only how but also when a drug or stressor is administered to the test subject.

An additional consideration exists regarding generational investigations on females of oviparous species [3]. In *Drosophila*, full maturation of reproductive organs first occurs after pupation, which means oogenesis only happens in the adult flies [18]. Exposure of adult females thus can affect mature oocytes, which include the germ plasm destined to form the second-generation offspring [18,77,78,79]. This puts a stressor in direct contact with F2 cells if the exposed oocyte contributed to establishing the next generation. This is equivalent to exposure of fetus germ cells within pregnant mammals [3]. The distinction between inter- and trans-generational effects can be ensured by not establishing an F1-offspring generation until the new oocytes are developed in the F0 female. Waiting 3–4 days before oviposition, as done in this study, allows for the production of a new batch of unexposed oocytes [80].

## 5. Conclusions

Our investigation highlights the importance of methodological awareness and of recognizing the physiological characteristics of model organisms. Environmental temperature, exposure timing and duration, sex, and genetic background all shaped the outcomes of paracetamol exposure in *Drosophila*. These factors can lead to divergent results across studies that differ in design, and they complicate direct comparisons between investigations. At the same time, they emphasize that variation in biological responses is not noise but information: it reflects the interaction between developmental timing, physiology, and genotype. Rather than diminishing the value of model systems, this complexity underscores the need for deliberate experimental choices and transparent reporting. Only by accounting for these biological and methodological variables can results be meaningfully integrated across studies and responsibly interpreted when considering broader toxicological questions. Awareness of how—and when—exposure occurs is, therefore, essential for both experimental design and the cautious extrapolation of findings beyond the model species.

## Figures and Tables

**Table 1:** Statistical overview for the dose–response curve showing the analysis of deviance table with relevant main effects and interactions.

Analysis of Deviance, Type II Wald Tests			
	χ^2^	Df	*p*-Values
Dose	215.799	1	<0.0001
Temp	80.854	1	<0.0001
Line	134.069	4	<0.0001
Dose^2^	57.556	1	<0.0001
Dose:Temp	75.079	1	<0.0001
Dose:Line	45.973	4	<0.0001
Temp:Line	30.354	4	<0.0001
Temp:Dose^2^	46.556	1	<0.0001
Line:Dose^2^	12.861	4	0.0120
Dose:Temp:Line	38.299	4	<0.0001

**Table 2:** Statistical overview for the spontaneous locomotor activity showing the analysis of variance table for each of the four models using the type III Wald χ^2^-test. The binomial model analyzes data separated depending on whether movement is detected (active) or not (passive) per minute for individual flies. The continuous model concerns the analyses of distance moved among the active flies. Degrees of freedom, χ^2^, and *p*-values are presented. Females are presented on the left, while males are on the right.

Binomial Model					
		Females		Males	
	Df	χ^2^	*p*-Value	χ^2^	*p*-Value
(Intercept)	1	1.5629	0.2112	7.2685	0.007
Treat	4	11.0595	0.0259	23.6127	<0.0001
Gen	2	6.8508	0.0325	13.0792	0.0014
Line	4	69.0471	<0.0001	37.4067	<0.0001
Treat:Gen	8	72.6482	<0.0001	43.8073	<0.0001
Treat:Line	16	62.1307	<0.0001	59.997	<0.0001
Gen:Line	8	78.9793	<0.0001	137.3828	<0.0001
Continuous Model					
		Females (Beta)		Males (Linear)	
	Df	χ^2^	*p*-value	χ^2^	*p*-value
(Intercept)	1	268.212	<0.0001	85.339	<0.0001
Treat	4	30.353	<0.0001	9.6165	0.04741
Gen	2	28.553	<0.0001	41.5137	<0.0001
Line	4	79.603	<0.0001	96.5979	<0.0001
Treat:Gen	8	40.496	<0.0001	49.3691	<0.0001
Treat:Line	16	128.403	<0.0001	62.7841	<0.0001
Gen:Line	8	55.6	<0.0001	108.7938	<0.0001

**Table 3:** Overview of treatment responses in female spontaneous locomotor activity separated into overall and line-specific responses, showing distance moved per minute. Comparisons were made at the generation level for each model, and for each row the letters signify the treatment relationship within that line and generation combination for that model. The grid is visualized like a heat map, with lighter colors signifying low activity and darker colors signifying high activity. Treatments that differ from the control of their grouping have further been bolded.

Female Spontaneous Locomotor Activity											
		Chance of Movement (Binomial Regression)					Length of Movement (Beta Regression)				
Gen	Line\Treat	C	J20	J40	A40	A80	C	J20	J40	A40	A80
F0	All	48.9% (bc)	64.3% (c)	**22.7% (a)**	29.2% (ab)	41.7% (abc)	0.112 (bc)	0.120 (c)	0.090 (ab)	**0.084 (a)**	0.109 (bc)
F1	All	25.3% (a)	**75.6% (bc)**	**87.7% (bc)**	**63.9% (b)**	**90.4 (c)**	0.097 (a)	**0.148 (b)**	**0.150 (b)**	0.106 (a)	**0.145 (b)**
F2	All	7.14% (a)	**34.5% (b)**	**41.1% (b)**	21.3% (ab)	12.1% (a)	0.070 (a)	**0.102 (c)**	**0.093 (bc)**	0.084 (abc)	0.076 (ab)
F0	L1	64.7% (ab)	81.3% (b)	48.7% (ab)	28.1% (a)	60.1% (ab)	0.145 (b)	0.152 (b)	0.123 (b)	**0.069 (a)**	0.128 (b)
	L2	6.92% (a)	17.4% (a)	12.2% (a)	3.87% (a)	21.7% (a)	0.079 (b)	0.077 (ab)	0.086 (b)	**0.050 (a)**	0.103 (ab)
	L3	81.1% (b)	83.4% (b)	**34.5% (a)**	**38.4% (a)**	54.1% (ab)	0.146 (b)	0.147 (b)	0.109 (ab)	**0.096 (a)**	0.102 (ab)
	L4	28.1% (b)	21.4% (ab)	**3.31% (a)**	48.2% (b)	25.7% (b)	0.069 (b)	0.077 (b)	**0.042 (a)**	**0.121 (c)**	0.078 (b)
	L5	77.8% (ab)	93.8% (b)	47.7% (a)	57.1% (a)	52.5% (a)	0.149 (bc)	0.179 (c)	0.114 (ab)	**0.102 (a)**	0.146 (bc)
F1	L1	28.1% (a)	**81.8% (b)**	**93.2% (b)**	50.2% (ab)	**92.3% (b)**	0.079 (ab)	**0.119 (c)**	0.129 (bc)	0.053 (a)	0.108 (bc)
	L2	9.0% (a)	**57.6% (bc)**	**92.7% (cd)**	39.1% (ab)	**93.2% (d)**	0.101 (a)	0.140 (ab)	**0.207 (b)**	0.094 (a)	**0.198 (b)**
	L3	51.5% (a)	**85.8 (b)**	89.9% (ab)	65.2% (ab)	**91.6% (b)**	0.127 (a)	**0.181 (b)**	0.180 (ab)	0.121 (a)	0.136 (ab)
	L4	5% (a)	15.1% (ab)	**23.9 (bc)**	**60.1% (c)**	**63.3% (c)**	0.060 (a)	**0.097 (b)**	0.074 (ab)	**0.151 (c)**	**0.105 (b)**
	L5	67.5% (a)	**97.7% (b)**	**97.4% (b)**	90.5% (ab)	**96.0% (b)**	0.139 (a)	**0.231 (b)**	**0.200 (b)**	0.137 (a)	**0.204 (b)**
F2	L1	54.8% (a)	**91.2% (bc)**	**94.9% (c)**	67.8% (abc)	72.3% (ab)	0.095 (ab)	**0.133 (c)**	0.131 (bc)	0.071 (a)	0.072 (ab)
	L2	2.26% (a)	**19.2% (bc)**	**56.1% (c)**	9.2% (ab)	**18.5% (bc)**	0.063 (a)	**0.084 (b)**	**0.116 (b)**	0.066 (a)	0.094 (ab)
	L3	12.5% (a)	37.9% (a)	34.2% (a)	14.5% (a)	9.36% (a)	0.08 (ab)	0.110 (b)	0.099 (ab)	0.085 (ab)	0.061 (a)
	L4	0.84% (a)	2.11% (a)	2.15% (a)	**14.2% (b)**	1.93% (a)	0.046 (a)	0.070 (a)	0.048 (a)	**0.130 (b)**	0.058 (a)
	L5	7.35% (a)	**55.3% (b)**	**37.9% (b)**	19.7% (ab)	6.15% (a)	0.074 (a)	**0.123 (b)**	0.094 (ab)	0.081 (a)	0.081 (a)

**Table 4:** Overview of treatment responses in male spontaneous locomotor activity separated into overall and line-specific responses, showing distance moved per minute. Comparisons were made at the generation level for each model, and for each row the letters signify the treatment relationship within that line and generation combination for that model. The grid is visualized like a heat map, with lighter colors signifying low activity and darker colors signifying high activity. Treatments that differ from the control of their grouping have further been bolded.

Male Spontaneous Locomotor Activity											
		Chance of Movement (Binomial Regression)					Length of Movement (Beta Regression)				
Gen	Line\Treat	C	J20	J40	A40	A80	C	J20	J40	A40	A80
F0	All	84.3% (a)	**96.3% (b)**	92% (ab)	**94.5% (b)**	**94.9% (b)**	0.152 (a)	0.167 (a)	0.143 (a)	0.144 (a)	0.171 (a)
F1	All	85.1% (b)	91.9% (b)	93.0% (bc)	**53.8% (a)**	**97.6% (c)**	0.158 (ab)	0.184 (bc)	**0.195 (c)**	0.127 (a)	**0.193 (c)**
F2	All	81.2% (b)	84.4% (bc)	**92.6% (cd)**	**51.7% (a)**	**96.6% (d)**	0.194 (c)	**0.144 (b)**	**0.157 (b)**	**0.090 (a)**	0.190 (c)
F0	L1	79.5% (a)	**98.3% (b)**	**98.7% (b)**	95.5% (ab)	97.1% (b)	0.143 (a)	0.191 (a)	0.141 (a)	0.129 (a)	0.180 (a)
	L2	42.7% (a)	60.2% (a)	17.3% (a)	53.3% (a)	52.7% (a)	0.079 (a)	0.077 (a)	0.039 (a)	0.082 (a)	0.058 (a)
	L3	91.1% (a)	97.5% (ab)	86.0% (a)	91.5% (ab)	**98.8% (b)**	0.147 (ab)	0.182 (bc)	0.141 (ab)	0.104 (a)	**0.218 (c)**
	L4	85.1% (a)	96.7% (a)	88.6% (a)	96.3% (a)	92.3% (a)	0.148 (a)	0.149 (a)	0.122 (a)	0.155 (a)	0.126 (a)
	L5	96.3% (a)	99.1% (ab)	**99.6% (b)**	**99.6% (b)**	98.4% (ab)	0.244 (a)	0.237 (a)	0.272 (a)	0.252 (a)	0.273 (a)
F1	L1	58.0% (a)	**89.5% (b)**	**96.6% (b)**	32.1% (a)	**96.1% (b)**	0.121 (ab)	0.179 (c)	0.165 (bc)	0.083 (a)	0.173 (bc)
	L2	51.5% (bc)	47.0% (bc)	24.4% (ab)	**9.37% (a)**	76.8% (c)	0.122 (a)	0.130 (a)	0.128 (a)	0.101 (a)	0.117 (a)
	L3	80.4% (b)	86.5% (b)	72.7% (b)	**21.4% (a)**	**98.5% (c)**	0.123 (b)	0.169 (bc)	0.163 (bc)	**0.056 (a)**	**0.209 (c)**
	L4	96.7% (ab)	98.4% (ab)	97.7% (ab)	89.5% (a)	99.2% (b)	0.172 (a)	0.183 (a)	0.191 (a)	0.156 (a)	0.165 (a)
	L5	97.1% (ab)	98.4% (abc)	**99.7% (c)**	94.9% (a)	99.4% (bc)	0.255 (ab)	0.258 (ab)	**0.329 (c)**	0.238 (a)	0.299 (bc)
F2	L1	91.7% (ab)	97.8% (bc)	**99.6% (c)**	82.3% (a)	**99.4% (c)**	0.224 (b)	0.207 (b)	0.195 (b)	**0.114 (a)**	0.239 (b)
	L2	91.6% (bc)	85.3% (abc)	80.4% (ab)	**56.4% (a)**	96.9% (c)	0.208 (b)	**0.141 (a)**	**0.140 (a)**	**0.115 (a)**	0.165 (ab)
	L3	48.9% (b)	48.6% (b)	43.5% (b)	**7.18% (a)**	**93.3% (c)**	0.138 (b)	0.109 (b)	0.105 (b)	**0.000 (a)**	**0.187 (c)**
	L4	60.0% (ab)	66.7% (ab)	73.1% (ab)	34.7% (a)	85.7% (b)	0.148 (b)	**0.084 (a)**	**0.094 (a)**	**0.058 (a)**	0.103 (ab)
	L5	89.5% (a)	90.8% (a)	**99.2% (b)**	85.1% (a)	97.4% (ab)	0.250 (b)	**0.178 (a)**	0.250 (b)	**0.161 (a)**	0.257 (b)

**Table 5:** Statistical overview for the spontaneous locomotor activity showing the analysis of variance table for each of the four models using the type III Wald χ^2^-test. Degrees of freedom, χ^2^, and *p*-values are presented. Females are presented on the left, while males are on the right.

		Females		Males	
	Df	χ^2^	*p*-Value	χ^2^	*p*-Value
Treat	4	12.312	0.0152	38.603	<0.0001
Gen	2	41.673	<0.0001	54.626	<0.0001
Line	4	164.57	<0.0001	163.902	<0.0001
Treat:Gen	8	74.726	<0.0001	133.243	<0.0001
Treat:Line	16	95.198	<0.0001	123.609	<0.0001
Gen:Line	8	178.793	<0.0001	120.605	<0.0001

**Table 6:** Overview of T_50_ from the starvation assay, showing the mean number of days before half the population died. Comparisons were made at the generation level for each sex, and for each row the letters signify the treatment relationship within that generation and sex combination. The grid is visualized like a heat map, with lighter colors signifying low starvation tolerance and darker colors signifying high starvation tolerance. Different letters signify post hoc comparison groupings for each generation or line (row within the table). Treatments that differ from the control group have further been boldened.

Starvation Tolerance						
Sex	Gen\Treat	C	J20	J40	A40	A80
Female	F0	54.5 (ab)	50.0 (ab)	46.3 (a)	59.4 (b)	50.5 (ab)
	F1	41.1 (ab)	48.4 (ab)	46.0 (b)	45.1 (b)	38.2 (a)
	F2	51.2 (a)	45.0 (a)	43.3 (a)	50.3 (a)	50.3 (a)
Male	F0	27.5 (a)	22.2 (a)	23.5 (a)	**33.7 (b)**	29.4 (ab)
	F1	29.0 (a)	29.3 (a)	31.1 (a)	30.0 (a)	28.5 (a)
	F2	26.1 (a)	25.8 (a)	26.0 (a)	24.4 (a)	27.8 (a)

## Data Availability

The raw data supporting the conclusions of this article will be made available by the authors upon request.

## Supplementary Materials

The following supporting information can be downloaded at: https://www.mdpi.com/article/10.3390/medicines13030024/s1. Figure S1: Activity comparison across generations for Line 1. Figure S2: Activity comparison across generations for Line 2. Figure S3: Activity comparison across generations for Line 3. Figure S4: Activity comparison across generations for Line 4. Figure S5: Activity comparison across generations for Line 5. Figure S6: Distance comparison across generations for Line 1. Figure S7: Distance comparison across generations for Line 2. Figure S8: Distance comparison across generations for Line 3. Figure S9: Distance comparison across generations for Line 4. Figure S10: Distance comparison across generations for Line 5. Table S1: Genotype and DGRP Stock numbers. Table S2: Statistical summary of dose–response model. Table S3: Statistical summary of the female activity model. Table S4: Female post hoc generational means and CIs for the activity model. Table S5: Female post hoc line means and CIs for the activity model. Table S6: Statistical summary of the male activity model. Table S7: Male post hoc generational means and CIs for the activity model. Table S8: Male post hoc line means and CIs for the activity model. Table S9: Statistical summary of the female distance model. Table S10: Female post hoc generational means and CIs for the distance model. Table S11: Female post hoc line means and CIs for the survival model. Table S12: Statistical summary of the male survival model. Table S13: Male post hoc generational means and CIs for the survival model. Table S14: Male post hoc line means and CIs for the survival model. Table S15: Full statistical overview of the female Weibull survival model for the starvation experiment, showing fixed effects, random effects, and sample size. Line 1, Generation F0, and the control treatment are used as the model baseline. Significant *p*-values are shown in bold. Table S16: Overview of means and confidence intervals averaged over lines in the post hoc analysis of the female Weibull survival model for the starvation experiment. Shows the mean, standard errors, 95% confidence interval, and groupings, as determined by treatment contrasts. Table S17: Overview of means and confidence intervals in the post hoc analysis of the female Weibull survival model for the starvation experiment. Shows the mean, standard errors, 95% confidence interval, and groupings, as determined by treatment contrasts. Table S18: Full statistical overview of the male lognormal survival model for the starvation experiment, showing fixed effects, random effects, and sample size. Line 1, Generation F0, and the control treatment are used as the model baseline. Significant *p*-values are shown in bold. Table S19: Overview of means and confidence intervals averaged over lines in the post hoc analysis of the male lognormal survival model for the starvation experiment. Shows the mean, standard errors, 95% confidence interval, and groupings, as determined by treatment contrasts. Table S20: Overview of means and confidence intervals in the post hoc analysis of the male lognormal survival model for the starvation experiment. Shows the mean, standard errors, 95% confidence interval, and groupings, as determined by treatment contrasts.
