# Supplementary material for: Paracetamol Responses Depend on Temperature, Life Stage, and Genetic Background in Drosophila melanogaster Across Generations
Source: Medicines (Basel). 2026 Jul 30;13(3):24. doi: 10.3390/medicines13030024 (PMC13511354; doi:10.3390/medicines13030024)

Supplement: Paracetamol Responses Depend on Temperature, Life Stage, and Genetic Background in Drosophila melanogaster Across Generations

Table S1 Genotype and Drosophila Genetic Reference Panel Stock numbers for the lines used in this investigation.

Line No.	Genotype	Stock
1	195	28153
2	379	25189
3	362	25187
4	517	25197
5	358	25185

Table S2 Full statistical overview of the dose-response curve, showing fixed effects, random effects, residuals. Line 1 and temperature 19 are used as the model baseline while dose is the a numerical value, scaled to ensure cleaner model behavior. Significant p-values are shown in bold.

Family: binomial (logit)				
Formula: outcome ~ scaled(Dose) * Temp * Line + Dose2 * Temp + scaled(Dose)2 * Line + (1 | Vial_ID)				
Fixed effects	Estimate	Std. Error	z value	P-value
(Intercept)	-1.480	0.233	-6.340	**0.0000**
Dose	-4.107	0.510	-8.046	**0.0000**
Temp25	0.886	0.300	2.954	**0.0031**
LineL2	1.154	0.319	3.620	**0.0003**
LineL3	-2.789	0.566	-4.925	**0.0000**
LineL4	0.286	0.357	0.803	0.4223
LineL5	-0.610	0.376	-1.623	0.1046
$Dose^2$	-3.584	0.550	-6.516	**0.0000**
Dose:Temp25	3.252	0.538	6.046	**0.0000**
Dose:LineL2	-0.957	0.572	-1.673	0.0943
Dose:LineL3	-6.060	0.890	-6.809	**0.0000**
Dose:LineL4	-2.784	0.645	-4.319	**0.0000**
Dose:LineL5	-1.132	0.650	-1.744	0.0812
Temp25:LineL2	0.683	0.414	1.648	0.0993
Temp25:LineL3	4.499	0.661	6.803	**0.0000**
Temp25:LineL4	0.106	0.453	0.235	0.8144
Temp25:LineL5	0.971	0.471	2.061	**0.0393**
Temp25:$Dose^2$	3.460	0.507	6.823	**0.0000**
LineL2:$Dose^2$	-1.156	0.452	-2.557	**0.0105**
LineL3:$Dose^2$	-1.353	0.468	-2.891	**0.0038**
LineL4:$Dose^2$	-0.777	0.479	-1.624	0.1045
LineL5:$Dose^2$	-0.217	0.449	-0.482	0.6297
Dose:Temp25:LineL2	0.514	0.618	0.832	0.4057
Dose:Temp25:LineL3	5.456	0.910	5.995	**0.0000**
Dose:Temp25:LineL4	1.458	0.670	2.177	**0.0295**
Dose:Temp25:LineL5	1.516	0.687	2.206	**0.0274**
Residuals				
Min	1Q	Median	3Q	Max
-2.577	-0.680	-0.0063	0.626	7.241

Random effect			
Groups	Name	Variance	Std.Dev.
Vial_ID	(Intercept)	0.245	0.495
Number of obs.: 10450, groups: Vial_ID, 209			

Table S3 Full statistical overview of the female Binomial model for the Spontaneous Locomotor Activity experiment, showing fixed effects, random effects, and sample size. Line 1, Generation F0 and the control treatment are used as the model baseline. Significant p-values are shown in bold.

Female Activity Model				
Family: binomial (logit)				
Active ~ Treat * Gen + Treat * Line + Line * Gen + (1 | ID)				
Fixed effects	Estimate	Std. Error	z value	P-value
(Intercept)	0.60749	0.48592	1.25	0.2112
TreatJ20	0.85926	0.60473	1.421	0.1553
TreatJ40	-0.65828	0.7179	-0.917	0.3592
TreatA40	-1.5473	0.73611	-2.102	**0.0356**
TreatA80	-0.19822	0.66499	-0.298	0.7656
GenF1	-1.54503	0.60315	-2.562	**0.0104**
GenF2	-0.41619	0.55871	-0.745	0.4563
LineL2	-3.20689	0.62957	-5.094	**< 0.0001**
LineL3	0.84894	0.63182	1.344	0.1791
LineL4	-1.54896	0.58818	-2.633	**0.0085**
LineL5	0.64851	0.56586	1.146	0.2518
TreatJ20:GenF1	1.57982	0.54386	2.905	**0.0037**
TreatJ40:GenF1	4.22776	0.76913	5.497	**< 0.0001**
TreatA40:GenF1	2.49275	0.61065	4.082	**< 0.0001**
TreatA80:GenF1	3.61421	0.64069	5.641	**< 0.0001**
TreatJ20:GenF2	1.29126	0.56087	2.302	0.0213
TreatJ40:GenF2	3.38635	0.58291	5.809	**< 0.0001**
TreatA40:GenF2	2.10094	0.61335	3.425	**0.0006**
TreatA80:GenF2	0.96631	0.57396	1.684	0.0923
TreatJ20:LineL2	0.17919	0.74721	0.24	0.8105
TreatJ40:LineL2	1.28242	0.95902	1.337	0.1811
TreatA40:LineL2	0.92285	0.95994	0.961	0.3364
TreatA80:LineL2	1.51416	0.84168	1.799	0.0720
TreatJ20:LineL3	-0.69885	0.7114	-0.982	0.3259
TreatJ40:LineL3	-1.44059	0.85949	-1.676	0.0937
TreatA40:LineL3	-0.3809	0.93876	-0.406	0.6849
TreatA80:LineL3	-1.09472	0.8218	-1.332	0.1828
TreatJ20:LineL4	-1.21721	0.73157	-1.664	0.0961
TreatJ40:LineL4	-1.77547	0.89371	-1.987	0.0470
TreatA40:LineL4	2.41589	0.88406	2.733	**0.0063**
TreatA80:LineL4	0.07773	0.76466	0.102	0.9190
TreatJ20:LineL5	0.59767	0.6998	0.854	0.3931
TreatJ40:LineL5	-0.6893	0.79514	-0.867	0.3860
TreatA40:LineL5	0.57595	0.87547	0.658	0.5106
TreatA80:LineL5	-0.95897	0.70999	-1.351	0.1768
GenF1:LineL2	1.83174	0.81816	2.239	**0.0252**
GenF2:LineL2	-0.74998	0.64749	-1.158	0.2467
GenF1:LineL3	0.15056	0.74356	0.202	0.8395

Female Activity Model				
Family: binomial (logit)				
Active ~ Treat * Gen + Treat * Line + Line * Gen + (1 | ID)				
Fixed effects	Estimate	Std. Error	z value	P-value
GenF2:LineL3	-2.98394	0.63767	-4.679	< **0.0001**
GenF1:LineL4	-0.46405	0.66893	-0.694	0.4879
GenF2:LineL4	-3.41433	0.67901	-5.028	< **0.0001**
GenF1:LineL5	1.02143	0.6642	1.538	0.1241
GenF2:LineL5	-3.37341	0.60284	-5.596	< **0.0001**

Random effect			
Groups	Name	Variance	Std.Dev.
ID	(Intercept)	6.174	2.485
Number of obs.: 41512, groups: ID, 1182			

Table S4 Overview of means and confidence interval averaged over lines in the post hoc analysis of the female Binomial model for the Spontaneous Locomotor Activity experiment. Shows the mean, standard errors, 95%-confidence interval and groupings as determined by treatment contrasts.

Generation	Treatment	Probability	Std. Error	95% CI	Group
F0	C	0.488953584	0.059141678	[0.3421; 0.6377]	bc
	J20	0.642729152	0.068456253	[0.4550; 0.7950]	c
	J40	0.226696534	0.059472387	[0.1090; 0.4126]	a
	A40	0.29219702	0.055775435	[0.1709; 0.4526]	ab
	A80	0.417081129	0.074800818	[0.2447; 0.6125]	abc
F1	C	0.253262497	0.048678981	[0.1488; 0.3969]	a
	J20	0.755830965	0.054017982	[0.5929; 0.8681]	bc
	J40	0.876922762	0.064341151	[0.6054; 0.9707]	bc
	A40	0.638973864	0.10267611	[0.3600; 0.8478]	b
	A80	0.903988808	0.039002588	[0.7474; 0.9677]	c
F2	C	0.071444357	0.01777122	[0.0372; 0.1330]	a
	J20	0.344788029	0.070694609	[0.1903; 0.5409]	b
	J40	0.41066268	0.076621599	[0.2356; 0.6117]	b
	A40	0.213439382	0.069383258	[0.0856; 0.4403]	ab
	A80	0.131361089	0.037484772	[0.0609; 0.2606]	a

Table S5 Overview of means and confidence interval in the post hoc analysis of the female Binomial model for the Spontaneous Locomotor Activity. Shows the mean, standard errors, 95%-confidence interval and groupings as determined by treatment contrasts.

Generation	Line	Treatment	Probability	Std. Error	95% CI	Group
F0	L1	C	0.64736748	0.11092765	[0.3443; 0.8652]	ab
		J20	0.81256284	0.07820542	[0.5360; 0.9421]	b
		J40	0.48730493	0.15740609	[0.1579; 0.8281]	ab
		A40	0.28093789	0.11566334	[0.0821; 0.6306]	a
		A80	0.60091311	0.14877301	[0.2335; 0.8816]	ab
	L2	C	0.06917712	0.02879962	[0.0229; 0.1904]	a
		J20	0.17351142	0.07846206	[0.0488; 0.4622]	a
		J40	0.12182566	0.06674529	[0.0271; 0.4090]	a
		A40	0.03827797	0.02031967	[0.0095; 0.1416]	a
		A80	0.21696308	0.09240176	[0.0639; 0.5293]	a
	L3	C	0.81098583	0.07342192	[0.5554; 0.9364]	b
		J20	0.83435868	0.0688018	[0.5829; 0.9478]	b
		J40	0.34469582	0.12543549	[0.1118; 0.6874]	a
		A40	0.38419621	0.1220566	[0.1418; 0.7021]	a
		A80	0.54078299	0.13262741	[0.2293; 0.8233]	ab
	L4	C	0.28060265	0.07799046	[0.1260; 0.5134]	b
		J20	0.21426173	0.08298756	[0.0712; 0.4926]	ab
		J40	0.03307876	0.02161831	[0.0060; 0.1633]	a
		A40	0.48178593	0.11453468	[0.2219; 0.7519]	b
		A80	0.25693491	0.10117241	[0.0811; 0.5752]	b
	L5	C	0.77833549	0.06265388	[0.5795; 0.8995]	ab
		J20	0.93778509	0.02999583	[0.8004; 0.9827]	b
		J40	0.47711894	0.12278039	[0.2044; 0.7642]	a
		A40	0.57068368	0.11835264	[0.2769; 0.8219]	a
		A80	0.52468261	0.12070076	[0.2409; 0.7934]	a
F1	L1	C	0.28139738	0.10202738	[0.0965; 0.5895]	a
		J20	0.81780457	0.07524341	[0.5500; 0.9428]	b
		J40	0.93288917	0.05929285	[0.5480; 0.9938]	b
		A40	0.50197565	0.23293436	[0.0838; 0.9174]	ab
		A80	0.92261769	0.03853642	[0.7480; 0.9795]	b
	L2	C	0.09007726	0.04723964	[0.0219; 0.3040]	a
		J20	0.57580757	0.13859007	[0.2394; 0.8541]	bc
		J40	0.9268456	0.07290836	[0.4426; 0.9951]	cd
		A40	0.39069329	0.21880123	[0.0567; 0.8725]	ab
		A80	0.93197737	0.058564	[0.5592; 0.9933]	d
	L3	C	0.51548451	0.12299207	[0.2303; 0.7909]	a
		J20	0.85841532	0.06254491	[0.6170; 0.9580]	b
		J40	0.89942505	0.08394514	[0.4503; 0.9899]	ab
		A40	0.65169527	0.12113799	[0.3212; 0.8809]	ab
		A80	0.91554018	0.06554524	[0.5498; 0.9897]	b
	L4	C	0.04971032	0.02039412	[0.0169; 0.1372]	a
		J20	0.15075607	0.06482696	[0.0460; 0.3955]	ab

Generation	Line	Treatment	Probability	Std. Error	95% CI	Group
F1	L4	J40	0.2392956	0.09204095	[0.0788; 0.5364]	bc
		A40	0.60127405	0.12055128	[0.2923; 0.8463]	c
		A80	0.63255021	0.12086967	[0.3108; 0.8679]	c
	L5	C	0.67533018	0.08332234	[0.4387; 0.8470]	a
		J20	0.9774486	0.01178814	[0.9162; 0.9942]	b
		J40	0.97372658	0.0154795	[0.8864; 0.9944]	b
		A40	0.90497665	0.0442759	[0.7166; 0.9729]	ab
		A80	0.96043438	0.02049336	[0.8582; 0.9898]	b
F2	L1	C	0.54767953	0.11805469	[0.2619; 0.8052]	a
		J20	0.91228198	0.0427148	[0.7245; 0.9763]	bc
		J40	0.94879579	0.02662987	[0.8187; 0.9870]	c
		A40	0.67807444	0.18414566	[0.1934; 0.9487]	abc
		A80	0.72299989	0.10053993	[0.4173; 0.9049]	ab
	L2	C	0.02263059	0.01185127	[0.0058; 0.0843]	a
		J20	0.19218948	0.08033626	[0.0590; 0.4743]	bc
		J40	0.56092912	0.17671621	[0.1675; 0.8902]	c
		A40	0.0920318	0.04582799	[0.0241; 0.2939]	ab
		A80	0.18492831	0.08199822	[0.0529; 0.4795]	bc
	L3	C	0.12524222	0.04431664	[0.0481; 0.2887]	a
		J20	0.37941616	0.11654942	[0.1459; 0.6863]	a
		J40	0.34159106	0.12327082	[0.1122; 0.6804]	a
		A40	0.14542274	0.09419838	[0.0236; 0.5452]	a
		A80	0.09361084	0.04841279	[0.0232; 0.3099]	a
	L4	C	0.00839247	0.00447065	[0.0021; 0.0327]	a
		J20	0.02106848	0.01299644	[0.0042; 0.0984]	a
		J40	0.02147004	0.01211047	[0.0049; 0.0883]	a
		A40	0.14155093	0.06507128	[0.0399; 0.3958]	b
		A80	0.01933771	0.01283312	[0.0034; 0.1013]	a
	L5	C	0.07353573	0.0266153	[0.0282; 0.1784]	a
		J20	0.5534425	0.11982155	[0.2623; 0.8121]	b
		J40	0.37875555	0.11433982	[0.1485; 0.6807]	b
		A40	0.19717953	0.08201513	[0.0607; 0.4826]	ab
		A80	0.06154434	0.0301241	[0.0168; 0.2008]	a

Table S6 Full statistical overview of the male Binomial model for the Spontaneous Locomotor Activity experiment, showing fixed effects, random effects, and sample size. Line 1, Generation F0 and the control treatment are used as the model baseline. Significant p-values are shown in bold.

Male Activity Model				
Family: binomial (logit)				
Active ~ Treat * Gen + Treat * Line + Line * Gen + (1 | ID)				
Fixed effects	Estimate	Std. Error	z value	P-value
(Intercept)	1.35989	0.50441	2.696	**0.0070**
TreatJ20	2.70596	0.78085	3.465	**0.0005**
TreatJ40	2.94704	0.70576	4.176	**0.0000**
TreatA40	1.68379	0.70432	2.391	**0.0168**
TreatA80	2.14262	0.70777	3.027	**0.0025**
GenF1	-1.03879	0.61646	-1.685	0.0920
GenF2	1.04895	0.5893	1.78	0.0751
LineL2	-1.65448	0.67324	-2.457	**0.0140**
LineL3	0.96801	0.66679	1.452	0.1466
LineL4	0.38494	0.60021	0.641	0.5213
LineL5	1.9007	0.60236	3.155	**0.0016**
TreatJ20:GenF1	-0.8883	0.64285	-1.382	0.1670
TreatJ40:GenF1	0.08025	0.6157	0.13	0.8963
TreatA40:GenF1	-2.7542	0.61206	-4.5	**< 0.0001**
TreatA80:GenF1	0.73231	0.59839	1.224	0.2210
TreatJ20:GenF2	-1.33985	0.60517	-2.214	**0.0268**
TreatJ40:GenF2	0.29418	0.59769	0.492	0.6226
TreatA40:GenF2	-2.55565	0.62748	-4.073	**< 0.0001**
TreatA80:GenF2	0.63868	0.60497	1.056	0.2911
TreatJ20:LineL2	-1.99867	0.85992	-2.324	**0.0201**
TreatJ40:LineL2	-4.21915	0.81204	-5.196	**< 0.0001**
TreatA40:LineL2	-1.25873	0.85954	-1.464	0.1431
TreatA80:LineL2	-1.73996	0.80539	-2.16	**0.0307**
TreatJ20:LineL3	-1.37651	0.80141	-1.718	0.0859
TreatJ40:LineL3	-3.45915	0.78057	-4.432	**< 0.0001**
TreatA40:LineL3	-1.64132	0.90111	-1.821	0.0685
TreatA80:LineL3	-0.09626	0.80602	-0.119	0.9049
TreatJ20:LineL4	-1.07763	0.79736	-1.351	0.1765
TreatJ40:LineL4	-2.64609	0.80073	-3.305	**0.0010**
TreatA40:LineL4	-0.16794	0.86856	-0.193	0.8467
TreatA80:LineL4	-1.39815	0.79969	-1.748	0.0804
TreatJ20:LineL5	-1.21829	0.83864	-1.453	0.1463
TreatJ40:LineL5	-0.57508	0.80222	-0.717	0.4735
TreatA40:LineL5	0.47109	0.81377	0.579	0.5627
TreatA80:LineL5	-1.28785	0.80282	-1.604	0.1087
GenF1:LineL2	1.39311	0.71286	1.954	0.0507
GenF2:LineL2	1.63254	0.68075	2.398	**0.0165**
GenF1:LineL3	0.12386	0.7198	0.172	0.8634

Male Activity Model				
Family: binomial (logit)				
Active ~ Treat * Gen + Treat * Line + Line * Gen + (1 | ID)				
Fixed effects	Estimate	Std. Error	z value	P-value
GenF2:LineL3	-3.42235	0.67957	-5.036	**< 0.0001**
GenF1:LineL4	2.67836	0.69674	3.844	**0.0001**
GenF2:LineL4	-2.38719	0.67918	-3.515	**0.0004**
GenF1:LineL5	1.30659	0.71834	1.819	0.0689
GenF2:LineL5	-2.16539	0.65577	-3.302	**0.0010**
Random effect				
Groups	Name	Variance	Std.Dev.	
ID	(Intercept)	6.617	2.572	
Number of obs.: 41512, groups: ID, 1182				

Table S7 Overview of means and confidence interval averaged over lines in the post hoc analysis of the male Binomial model for the Spontaneous Locomotor Activity experiment. Shows the mean, standard errors, 95%-confidence interval and groupings as determined by treatment contrasts.

Generation	Treatment	Probability	Std. Error	95% CI	Group
F0	C	0.842868818	0.034308858	[0.7335; 0.9127]	a
	J20	0.962726029	0.014336575	[0.9022; 0.9864]	b
	J40	0.920332366	0.026304119	[0.8209; 0.9668]	ab
	A40	0.945014965	0.016817074	[0.8819; 0.9753]	b
	A80	0.948724337	0.016795382	[0.8838; 0.9783]	b
F1	C	0.850855571	0.032913272	[0.7452; 0.9175]	b
	J20	0.918696855	0.025988824	[0.8218; 0.9651]	b
	J40	0.93013198	0.02252537	[0.8450; 0.9702]	bc
	A40	0.537811098	0.091877966	[0.3099; 0.7509]	a
	A80	0.976149472	0.007901205	[0.9447; 0.9899]	c
F2	C	0.811563951	0.038659652	[0.6919; 0.8920]	b
	J20	0.844499785	0.039508014	[0.7145; 0.9218]	bc
	J40	0.925637407	0.021558977	[0.8475; 0.9654]	cd
	A40	0.517234902	0.098755055	[0.2789; 0.7479]	a
	A80	0.965678468	0.011372203	[0.9208; 0.9855]	d

Table S8 Overview of means and confidence interval in the post hoc analysis of the male Binomial model for the Spontaneous Locomotor Activity. Shows the mean, standard errors, 95%-confidence interval and groupings as determined by treatment contrasts.

Generation	Line	Treatment	Probability	Std. Error	95% CI	Group
F0	L1	C	0.79574263	0.08198454	[0.5152; 0.9346]	a
F0	L1	J20	0.98314081	0.01295898	[0.8861; 0.9977]	b
F0	L1	J40	0.98670433	0.00862949	[0.9317; 0.9975]	b
F0	L1	A40	0.95450903	0.02533581	[0.8236; 0.9895]	ab
F0	L1	A80	0.97075916	0.01882206	[0.8575; 0.9946]	b
F0	L2	C	0.57959379	0.12133756	[0.2766; 0.8325]	a
F0	L2	J20	0.89461459	0.05253833	[0.6689; 0.9727]	b
F0	L2	J40	0.96605217	0.019045	[0.8644; 0.9922]	b
F0	L2	A40	0.32097199	0.18066553	[0.0529; 0.7999]	a
F0	L2	A80	0.96068474	0.02184047	[0.8464; 0.9909]	b
F0	L3	C	0.9174994	0.03774764	[0.7548; 0.9757]	ab
F0	L3	J20	0.9775763	0.01281688	[0.9063; 0.9949]	bc
F0	L3	J40	0.99649505	0.00197488	[0.9851; 0.9992]	c
F0	L3	A40	0.82302641	0.08451019	[0.5106; 0.9540]	a
F0	L3	A80	0.99445967	0.00326625	[0.9750; 0.9988]	c
F0	L4	C	0.4268825	0.12575709	[0.1654; 0.7368]	a
F0	L4	J20	0.60173852	0.15991835	[0.2131; 0.8939]	a
F0	L4	J40	0.17268837	0.07248683	[0.0535; 0.4354]	a
F0	L4	A40	0.5325717	0.13151177	[0.2261; 0.8163]	a
F0	L4	A80	0.52699127	0.13063398	[0.2241; 0.8112]	a
F0	L5	C	0.51493138	0.12134407	[0.2330; 0.7877]	bc
F0	L5	J20	0.4697222	0.13653535	[0.1775; 0.7843]	bc
F0	L5	J40	0.24377055	0.10225	[0.0717; 0.5736]	ab
F0	L5	A40	0.09368864	0.0440933	[0.0264; 0.2826]	a
F0	L5	A80	0.76758163	0.09112187	[0.4698; 0.9249]	c
F1	L1	C	0.91582377	0.03967864	[0.7429; 0.9762]	bc
F1	L1	J20	0.85250152	0.07199452	[0.5694; 0.9619]	abc
F1	L1	J40	0.80360572	0.08510942	[0.5050; 0.9426]	ab
F1	L1	A40	0.56373098	0.14394479	[0.2225; 0.8537]	a
F1	L1	A80	0.96857570	0.01576561	[0.8903; 0.9915]	c
F1	L2	C	0.91116202	0.04134053	[0.7335; 0.9745]	a
F1	L2	J20	0.97484843	0.01327972	[0.9057; 0.9936]	ab
F1	L2	J40	0.86005999	0.06248685	[0.6174; 0.9590]	a
F1	L2	A40	0.91454042	0.04340992	[0.7190; 0.9781]	ab
F1	L2	A80	0.98755929	0.00717774	[0.9463; 0.9972]	b
F1	L3	C	0.80423570	0.07639072	[0.5407; 0.9348]	b
F1	L3	J20	0.86461211	0.06037407	[0.6285; 0.9602]	b
F1	L3	J40	0.72732899	0.10447748	[0.4071; 0.9120]	b
F1	L3	A40	0.21437543	0.08933239	[0.0651; 0.5169]	a
F1	L3	A80	0.9851039	0.00923915	[0.9289; 0.9970]	c
F1	L4	C	0.48862929	0.09652857	[0.2610; 0.7210]	b
F1	L4	J20	0.48603212	0.12321606	[0.2098; 0.7711]	b

Generation	Line	Treatment	Probability	Std. Error	95% CI	Group
F1	L4	J40	0.43452197	0.12605829	[0.1701; 0.7423]	b
		A40	0.07184645	0.04911684	[0.0115; 0.3404]	a
		A80	0.93336378	0.02993466	[0.8022; 0.9798]	c
	L5	C	0.85130073	0.04862047	[0.6804; 0.9390]	a
		J20	0.96685548	0.01687286	[0.8826; 0.9912]	a
		J40	0.88552065	0.06443037	[0.6008; 0.9755]	a
		A40	0.96305534	0.02155967	[0.8455; 0.9920]	a
		A80	0.92338884	0.03872498	[0.7464; 0.9801]	a
F2	L1	C	0.96721378	0.01222464	[0.9162; 0.9876]	ab
		J20	0.98408491	0.00897487	[0.9339; 0.9963]	ab
		J40	0.97737032	0.01336631	[0.9011; 0.9951]	ab
		A40	0.89529989	0.05421916	[0.6584; 0.9743]	a
		A80	0.9923182	0.00429647	[0.9680; 0.9982]	b
	L2	C	0.60027317	0.1079893	[0.3202; 0.8272]	ab
		J20	0.66709722	0.1129399	[0.3510; 0.8813]	ab
		J40	0.73139842	0.10514141	[0.4069; 0.9153]	ab
		A40	0.34678683	0.15473152	[0.0837; 0.7551]	a
		A80	0.85689705	0.07766315	[0.5395; 0.9684]	b
	L3	C	0.96305189	0.0138835	[0.9051; 0.9862]	a
		J20	0.99140774	0.00505795	[0.9615; 0.9981]	ab
		J40	0.99643333	0.00217091	[0.9830; 0.9993]	b
		A40	0.99557247	0.00244933	[0.9817; 0.9989]	b
		A80	0.98394197	0.0089434	[0.9345; 0.9962]	ab
	L4	C	0.97148497	0.01273693	[0.9125; 0.9911]	ab
		J20	0.98413686	0.01076705	[0.9130; 0.9973]	abc
		J40	0.99747906	0.0015401	[0.9879; 0.9995]	c
		A40	0.94926513	0.0267456	[0.8174; 0.9874]	a
		A80	0.99403267	0.00332902	[0.9752; 0.9986]	bc
	L5	C	0.89512103	0.03806538	[0.7502; 0.9604]	a
		J20	0.90821004	0.04360251	[0.7201; 0.9744]	a
		J40	0.99192033	0.00455002	[0.9660; 0.9981]	b
		A40	0.8511169	0.0674818	[0.5919; 0.9575]	a
		A80	0.9743593	0.01546756	[0.8852; 0.9947]	ab

Table S9 Full statistical overview of the female Beta model for the Spontaneous Locomotor Activity experiment, showing fixed effects, random effects, and sample size. Line 1, Generation F0 and the control treatment are used as the model baseline. Significant p-values are shown in bold.

Female Distance Model				
Family: beta (logit)				
Distance ~ Treat * Gen + Treat * Line + Line * Gen + (1 | ID)				
Fixed effects	Estimate	Std. Error	z value	P-value
(Intercept)	-1.766561	0.107867	-16.377	< **0.0001**
TreatJ20	0.04783	0.136075	0.351	0.7252
TreatJ40	-0.201398	0.157818	-1.276	0.2019
TreatA40	-0.84339	0.168802	-4.996	< **0.0001**
TreatA80	-0.149228	0.150511	-0.991	0.3215
GenF1	-0.690502	0.136363	-5.064	< **0.0001**
GenF2	-0.489859	0.126424	-3.875	**0.0001**
LineL2	-0.692947	0.150234	-4.612	< **0.0001**
LineL3	-0.003754	0.139082	-0.027	0.9785
LineL4	-0.83233	0.139374	-5.972	< **0.0001**
LineL5	0.026523	0.125108	0.212	0.8321
TreatJ20:GenF1	0.404973	0.123859	3.27	**0.0011**
TreatJ40:GenF1	0.746546	0.176723	4.224	< **0.0001**
TreatA40:GenF1	0.417469	0.139716	2.988	**0.0028**
TreatA80:GenF1	0.490245	0.144883	3.384	**0.0007**
TreatJ20:GenF2	0.335586	0.134591	2.493	**0.0127**
TreatJ40:GenF2	0.561603	0.139966	4.012	**0.0001**
TreatA40:GenF2	0.52829	0.154613	3.417	**0.0006**
TreatA80:GenF2	0.121454	0.14352	0.846	0.3974
TreatJ20:LineL2	-0.074128	0.184431	-0.402	0.6877
TreatJ40:LineL2	0.303209	0.227819	1.331	0.1832
TreatA40:LineL2	0.355269	0.237655	1.495	0.1349
TreatA80:LineL2	0.449176	0.206344	2.177	**0.0295**
TreatJ20:LineL3	-0.037581	0.156389	-0.24	0.8101
TreatJ40:LineL3	-0.133965	0.192788	-0.695	0.4871
TreatA40:LineL3	0.374203	0.213144	1.756	0.0792
TreatA80:LineL3	-0.260137	0.192884	-1.349	0.1774
TreatJ20:LineL4	0.071958	0.180512	0.399	0.6902
TreatJ40:LineL4	-0.315893	0.218328	-1.447	0.1479
TreatA40:LineL4	1.455789	0.208523	6.981	**0.0001**
TreatA80:LineL4	0.27515	0.181214	1.518	0.1289
TreatJ20:LineL5	0.169252	0.150452	1.125	0.2606
TreatJ40:LineL5	-0.105766	0.176269	-0.6	0.5485
TreatA40:LineL5	0.410095	0.203066	2.02	**0.0434**
TreatA80:LineL5	0.121955	0.159697	0.764	0.4451
GenF1:LineL2	0.958694	0.201211	4.765	**0.0001**
GenF2:LineL2	0.256693	0.157646	1.628	0.1035
GenF1:LineL3	0.534087	0.164641	3.244	**0.0012**

Female Distance Model				
Family: beta (logit)				
Distance ~ Treat * Gen + Treat * Line + Line * Gen + (1 | ID)				
Fixed effects	Estimate	Std. Error	z value	P-value
GenF2:LineL3	-0.179913	0.144073	-1.249	0.2118
GenF1:LineL4	0.534926	0.156317	3.422	**0.0006**
GenF2:LineL4	0.04997	0.171568	0.291	0.7709
GenF1:LineL5	0.605989	0.144719	4.187	**0.0001**
GenF2:LineL5	-0.291365	0.135495	-2.15	**0.0315**

Random effect			
Groups	Name	Variance	Std.Dev.
ID	(Intercept)	0.2832	0.5322
Number of obs.: 18850, groups: ID, 1016			

Table S10 Overview of means and confidence interval averaged over lines in the post hoc analysis of the female Beta model for the Spontaneous Locomotor Activity experiment. Shows the mean, standard errors, 95%-confidence interval and groupings as determined by treatment contrasts.

Generation	Treatment	Mean	Std. Error	95% CI	Group
F0	C	0.11233963	0.005350606	[0.0993; 0.1269]	bc
	J20	0.119904759	0.007246309	[0.1025; 0.1399]	c
	J40	0.089566183	0.006761674	[0.0736; 0.1086]	ab
	A40	0.083832261	0.005006652	[0.0718; 0.0977]	a
	A80	0.109188086	0.007185033	[0.0920; 0.1291]	bc
F1	C	0.097016269	0.005730923	[0.0832; 0.1128]	a
	J20	0.147779679	0.00796981	[0.1284; 0.1695]	b
	J40	0.149801286	0.016987869	[0.1111; 0.1990]	b
	A40	0.105488515	0.00944787	[0.0835; 0.1324]	a
	A80	0.145221801	0.012211138	[0.1165; 0.1796]	b
F2	C	0.069794889	0.004660763	[0.0587; 0.0828]	a
	J20	0.101512764	0.007080486	[0.0847; 0.1213]	c
	J40	0.092782627	0.006502992	[0.0773; 0.1109]	bc
	A40	0.084256278	0.008064803	[0.0657; 0.1075]	abc
	A80	0.075830919	0.005883754	[0.0620; 0.0924]	ab

Table S11 Overview of means and confidence interval in the post hoc analysis of the female Beta model for the Spontaneous Locomotor Activity. Shows the mean, standard errors, 95%-confidence interval and groupings as determined by treatment contrasts.

Generation	Line	Treatment	Mean	Std. Error	95% CI	Group
F0	L1	C	0.14597053	0.01344706	[0.1146; 0.1841]	b
		J20	0.15203467	0.01509036	[0.1171; 0.1951]	b
		J40	0.1226083	0.01468796	[0.0895; 0.1657]	b
		A40	0.06850072	0.00853461	[0.0495; 0.0940]	a
		A80	0.12833192	0.01555736	[0.0933; 0.1740]	b
	L2	C	0.07874601	0.00815926	[0.0601; 0.1025]	b
		J20	0.07685925	0.01055609	[0.0537; 0.1088]	ab
		J40	0.08645592	0.01225454	[0.0597; 0.1237]	b
		A40	0.04984866	0.00696849	[0.0347; 0.0712]	a
		A80	0.10344129	0.01398396	[0.0726; 0.1454]	b
	L3	C	0.14550312	0.01291651	[0.1153; 0.1820]	b
		J20	0.14678201	0.01396224	[0.1143; 0.1865]	b
		J40	0.10854617	0.01346433	[0.0784; 0.1484]	ab
		A40	0.09625879	0.01008601	[0.0732; 0.1256]	a
		A80	0.10159009	0.01114044	[0.0763; 0.1341]	ab
	L4	C	0.06920985	0.00635677	[0.0545; 0.0875]	b
		J20	0.07733622	0.0084705	[0.0581; 0.1022]	b
		J40	0.04244471	0.0071648	[0.0274; 0.0652]	a
		A40	0.12062855	0.01143867	[0.0941; 0.1533]	c
		A80	0.07777509	0.00940082	[0.0568; 0.1057]	b
	L5	C	0.14930817	0.01027367	[0.1247; 0.1777]	bc
		J20	0.17902669	0.01496577	[0.1437; 0.2209]	c
		J40	0.11433544	0.01167073	[0.0875; 0.1480]	ab
		A40	0.10217091	0.01073103	[0.0777; 0.1333]	a
		A80	0.14587717	0.01356131	[0.1143; 0.1844]	bc
F1	L1	C	0.07892356	0.00850241	[0.0596; 0.1038]	ab
		J20	0.11875633	0.01143901	[0.0923; 0.1515]	c
		J40	0.12876588	0.02360613	[0.0792; 0.2026]	bc
		A40	0.05300115	0.01064624	[0.0314; 0.0881]	a
		A80	0.10754698	0.01076927	[0.0828; 0.1386]	bc
	L2	C	0.10053295	0.01453587	[0.0688; 0.1446]	a
		J20	0.14031918	0.01552543	[0.1049; 0.1853]	ab
		J40	0.2070238	0.04320664	[0.1170; 0.3396]	b
		A40	0.09432202	0.01982683	[0.0542; 0.1592]	a
		A80	0.19763791	0.03604945	[0.1206; 0.3067]	b
	L3	C	0.12711284	0.01230979	[0.0986; 0.1623]	a
		J20	0.18071525	0.01627283	[0.1425; 0.2265]	b
		J40	0.1801181	0.03173953	[0.1121; 0.2765]	ab
		A40	0.12148424	0.0126787	[0.0924; 0.1581]	a
		A80	0.13636073	0.02294063	[0.0873; 0.2068]	ab
	L4	C	0.05983485	0.00655433	[0.0450; 0.0791]	a
		J20	0.09711441	0.01170809	[0.0709; 0.1317]	b

Generation	Line	Treatment	Mean	Std. Error	95% CI	Group
F1	L4	J40	0.07410953	0.00825612	[0.0555; 0.0984]	ab
		A40	0.15127974	0.01422848	[0.1182; 0.1917]	c
		A80	0.10542964	0.01117185	[0.0799; 0.1379]	b
	L5	C	0.13888872	0.01020247	[0.1146; 0.1673]	a
		J20	0.23103154	0.01858021	[0.1867; 0.2823]	b
		J40	0.20018018	0.0196774	[0.1542; 0.2557]	b
		A40	0.13700675	0.01315866	[0.1065; 0.1746]	a
		A80	0.20398391	0.01817832	[0.1611; 0.2548]	b
F2	L1	C	0.09479715	0.00913295	[0.0737; 0.1211]	ab
		J20	0.13319454	0.01245687	[0.1042; 0.1687]	c
		J40	0.13053745	0.01306803	[0.1004; 0.1680]	bc
		A40	0.07099399	0.01319675	[0.0437; 0.1134]	a
		A80	0.09244056	0.00940849	[0.0709; 0.1197]	ab
	L2	C	0.06340704	0.00897605	[0.0439; 0.0908]	a
		J20	0.08444845	0.01013093	[0.0618; 0.1145]	ab
		J40	0.11616485	0.01844053	[0.0764; 0.1727]	b
		A40	0.06583475	0.00931671	[0.0455; 0.0943]	a
		A80	0.09353039	0.01074931	[0.0693; 0.1251]	ab
	L3	C	0.08016648	0.00767435	[0.0625; 0.1023]	ab
		J20	0.10965674	0.01087258	[0.0846; 0.1410]	b
		J40	0.09851386	0.01112265	[0.0733; 0.1311]	ab
		A40	0.08463424	0.01421459	[0.0545; 0.1292]	ab
		A80	0.06134132	0.0089394	[0.0420; 0.0888]	a
	L4	C	0.04570439	0.00646752	[0.0317; 0.0656]	a
		J20	0.07021412	0.01200211	[0.0449; 0.1081]	a
		J40	0.04767645	0.0076125	[0.0315; 0.0716]	a
		A40	0.13032479	0.01649784	[0.0934; 0.1790]	b
		A80	0.05779055	0.0094746	[0.0377; 0.0876]	a
	L5	C	0.07438105	0.00689126	[0.0585; 0.0942]	a
		J20	0.12254004	0.01256987	[0.0937; 0.1588]	b
		J40	0.09390817	0.00959359	[0.0719; 0.1217]	ab
		A40	0.08119152	0.00898659	[0.0609; 0.1076]	a
		A80	0.0811309	0.01004349	[0.0587; 0.1111]	a

Table S12 Full statistical overview of the male Linear model for the Spontaneous Locomotor Activity experiment, showing fixed effects, random effects, and sample size. Line 1, Generation F0 and the control treatment are used as the model baseline. Significant p-values are shown in bold.

Male Distance Model				
Family: Linear				
Distance ~ Treat * Gen + Treat * Line + Line * Gen + (1 | ID)				
Fixed effects	Estimate	Std. Error	z value	P-value
(Intercept)	0.142526	0.015428	9.238	< **0.0001**
TreatJ20	0.047978	0.022701	2.113	**0.0346**
TreatJ40	-0.001527	0.020388	-0.075	0.9403
TreatA40	-0.013815	0.021263	-0.65	0.5159
TreatA80	0.03713	0.02072	1.792	0.0731
GenF1	-0.021903	0.018623	-1.176	0.2395
GenF2	0.081197	0.017464	4.649	< **0.0001**
LineL2	-0.063489	0.021507	-2.952	**0.0032**
LineL3	0.004266	0.020063	0.213	0.8316
LineL4	0.005868	0.018285	0.321	0.7483
LineL5	0.101681	0.017728	5.736	< **0.0001**
TreatJ20:GenF1	0.010309	0.018459	0.558	0.5765
TreatJ40:GenF1	0.046053	0.018226	2.527	**0.0115**
TreatA40:GenF1	-0.024022	0.01845	-1.302	0.1929
TreatA80:GenF1	0.015306	0.017365	0.881	0.3781
TreatJ20:GenF2	-0.064774	0.017946	-3.609	**0.0003**
TreatJ40:GenF2	-0.027472	0.01776	-1.547	0.1219
TreatA40:GenF2	-0.096157	0.019097	-5.035	< **0.0001**
TreatA80:GenF2	-0.022239	0.017517	-1.27	0.2042
TreatJ20:LineL2	-0.050373	0.026699	-1.887	0.0592
TreatJ40:LineL2	-0.038777	0.025216	-1.538	0.1241
TreatA40:LineL2	0.017197	0.027111	0.634	0.5259
TreatA80:LineL2	-0.057734	0.024362	-2.37	**0.0178**
TreatJ20:LineL3	-0.012444	0.024122	-0.516	0.6059
TreatJ40:LineL3	-0.003977	0.023266	-0.171	0.8643
TreatA40:LineL3	-0.02919	0.027688	-1.054	0.2918
TreatA80:LineL3	0.03386	0.023314	1.452	0.1464
TreatJ20:LineL4	-0.047001	0.023311	-2.016	**0.0438**
TreatJ40:LineL4	-0.024959	0.023122	-1.079	0.2804
TreatA40:LineL4	0.020485	0.026018	0.787	0.4311
TreatA80:LineL4	-0.059286	0.023401	-2.533	**0.0113**
TreatJ20:LineL5	-0.055052	0.02402	-2.292	**0.0219**
TreatJ40:LineL5	0.029321	0.022045	1.33	0.1835
TreatA40:LineL5	0.021129	0.024298	0.87	0.3845
TreatA80:LineL5	-0.008093	0.022559	-0.359	0.7198
GenF1:LineL2	0.064852	0.022273	2.912	**0.0036**
GenF2:LineL2	0.047896	0.020638	2.321	**0.0203**
GenF1:LineL3	-0.002172	0.021284	-0.102	0.9187

Male Distance Model				
Family: Linear				
Distance ~ Treat * Gen + Treat * Line + Line * Gen + (1 | ID)				
Fixed effects	Estimate	Std. Error	z value	P-value
GenF2:LineL3	-0.089598	0.019892	-4.504	**< 0.0001**
GenF1:LineL4	0.045166	0.020394	2.215	**0.0268**
GenF2:LineL4	-0.081833	0.020213	-4.048	**0.0001**
GenF1:LineL5	0.032743	0.020501	1.597	0.1102
GenF2:LineL5	-0.075665	0.01863	-4.061	**< 0.0001**
Random effect				
Groups	Name	Variance	Std.Dev.	
ID	(Intercept)	0.006628	0.08141	
Residual		0.004568	0.06759	
Number of obs.: 32876, groups: ID, 1238				

Table S13 Overview of means and confidence interval averaged over lines in the post hoc analysis of the male Linear model for the Spontaneous Locomotor Activity experiment. Shows the mean, standard errors, 95%-confidence interval and groupings as determined by treatment contrasts.

Generation	Treatment	Mean	Std. Error	95% CI	Group
F0	C	0.152190894	0.007978928	[0.1316; 0.1727]	a
	J20	0.16719475	0.011253756	[0.1382; 0.1962]	a
	J40	0.14298565	0.01047269	[0.1160; 0.1700]	a
	A40	0.144300402	0.009277635	[0.1204; 0.1682]	a
	A80	0.171070101	0.00980048	[0.1458; 0.1963]	a
F1	C	0.158405661	0.007789933	[0.1383; 0.1785]	ab
	J20	0.18371853	0.010086035	[0.1577; 0.2097]	bc
	J40	0.195253536	0.009917055	[0.1697; 0.2208]	c
	A40	0.126493461	0.011699325	[0.0964; 0.1566]	a
	A80	0.192590447	0.009322238	[0.1686; 0.2166]	c
F2	C	0.193548093	0.007834958	[0.1734; 0.2137]	c
	J20	0.143777508	0.009048692	[0.1205; 0.1671]	b
	J40	0.156871224	0.009147046	[0.1333; 0.1804]	b
	A40	0.089500176	0.012610325	[0.0570; 0.1220]	a
	A80	0.190188065	0.00940576	[0.1660; 0.2144]	c

Table S14 Overview of means and confidence interval in the post hoc analysis of the male Linear model for the Spontaneous Locomotor Activity. Shows the mean, standard errors, 95%-confidence interval and groupings as determined by treatment contrasts.

Generation	Line	Treatment	Mean	Std. Error	95% CI	Group
F0	L1	C	0.14252561	0.01542834	[0.1028; 0.1823]	a
		J20	0.19050327	0.02222458	[0.1333; 0.2478]	a
		J40	0.14099896	0.01836953	[0.0937; 0.1883]	a
		A40	0.12871078	0.01715769	[0.0845; 0.1729]	a
		A80	0.17965544	0.01916277	[0.1303; 0.2290]	a
	L2	C	0.07903691	0.01706843	[0.0351; 0.1230]	a
		J20	0.07664191	0.02073871	[0.0232; 0.1301]	a
		J40	0.03873282	0.01657198	[-0.004; 0.0814]	a
		A40	0.08241945	0.01701954	[0.0386; 0.1263]	a
		A80	0.05843258	0.01637066	[0.0163; 0.1006]	a
	L3	C	0.14679167	0.01516491	[0.1077; 0.1859]	ab
		J20	0.18232559	0.01573409	[0.1418; 0.2229]	bc
		J40	0.14128761	0.01577871	[0.1006; 0.1819]	ab
		A40	0.10378679	0.01704998	[0.0599; 0.1477]	a
		A80	0.2177813	0.01523401	[0.1785; 0.2570]	c
	L4	C	0.14839361	0.01179687	[0.1180; 0.1788]	a
		J20	0.14937041	0.01536581	[0.1098; 0.1890]	a
		J40	0.12190776	0.01912248	[0.0727; 0.1712]	a
		A40	0.15506404	0.01684087	[0.1117; 0.1984]	a
		A80	0.1262372	0.01607522	[0.0848; 0.1676]	a
	L5	C	0.24420668	0.01087693	[0.2162; 0.2722]	a
		J20	0.23713257	0.01550906	[0.1972; 0.2771]	a
		J40	0.27200111	0.01582956	[0.2312; 0.3128]	a
		A40	0.25152096	0.01536343	[0.2119; 0.2911]	a
		A80	0.27324399	0.01534331	[0.2337; 0.3128]	a
F1	L1	C	0.12062268	0.01561824	[0.0804; 0.1609]	ab
		J20	0.17890937	0.01705095	[0.1350; 0.2228]	c
		J40	0.16514916	0.01598134	[0.1240; 0.2063]	bc
		A40	0.08278615	0.02508014	[0.0182; 0.1474]	a
		A80	0.1730581	0.01660489	[0.1303; 0.2158]	bc
	L2	C	0.12198629	0.01537498	[0.0824; 0.1616]	a
		J20	0.1299003	0.01845561	[0.0824; 0.1774]	a
		J40	0.12773532	0.01953855	[0.0774; 0.1781]	a
		A40	0.10134712	0.01754128	[0.0562; 0.1465]	a
		A80	0.11668754	0.01543383	[0.0769; 0.1564]	a
	L3	C	0.12271648	0.01465335	[0.0850; 0.1605]	b
		J20	0.16855941	0.01558631	[0.1284; 0.2087]	bc
		J40	0.16326554	0.01678559	[0.1200; 0.2065]	bc
		A40	0.05568989	0.01725353	[0.0112; 0.1001]	a
		A80	0.20901169	0.01663321	[0.1662; 0.2519]	c
	L4	C	0.17165641	0.01066007	[0.1442; 0.1991]	a
		J20	0.18294223	0.01518508	[0.1438; 0.2221]	a

Generation	Line	Treatment	Mean	Std. Error	95% CI	Group
F1	L4	J40	0.19122367	0.01566426	[0.1509; 0.2316]	a
		A40	0.15430513	0.01797178	[0.1080; 0.2006]	a
		A80	0.16480558	0.01599765	[0.1236; 0.2060]	a
	L5	C	0.25504644	0.01304436	[0.2214; 0.2886]	ab
		J20	0.25828135	0.01818553	[0.2114; 0.3051]	ab
		J40	0.32889399	0.01516966	[0.2898; 0.3680]	c
		A40	0.23833902	0.01562492	[0.1981; 0.2786]	a
		A80	0.29938933	0.01509591	[0.2605; 0.3383]	bc
F2	L1	C	0.22372266	0.01489571	[0.1854; 0.2621]	b
		J20	0.20692589	0.01607583	[0.1655; 0.2483]	b
		J40	0.19472439	0.01541490	[0.1550; 0.2344]	b
		A40	0.11375041	0.01787853	[0.0677; 0.1598]	a
		A80	0.23861326	0.01559815	[0.1984; 0.2788]	b
	L2	C	0.20813035	0.01629072	[0.1662; 0.2501]	b
		J20	0.14096091	0.01744591	[0.0960; 0.1859]	a
		J40	0.14035463	0.01669663	[0.0973; 0.1834]	a
		A40	0.11535546	0.01899823	[0.0664; 0.1643]	a
		A80	0.16528678	0.01543754	[0.1255; 0.2051]	ab
	L3	C	0.13839029	0.012629	[0.1059; 0.1709]	b
		J20	0.10914976	0.01523704	[0.0699; 0.1484]	b
		J40	0.1054146	0.01556186	[0.0653; 0.1455]	b
		A40	-0.000772	0.02301193	[-0.06; 0.0585]	a
		A80	0.18714068	0.01452835	[0.1497; 0.2246]	c
	L4	C	0.14775801	0.01388523	[0.1120; 0.1835]	b
		J20	0.08396038	0.01558775	[0.0438; 0.1241]	a
		J40	0.09380054	0.01690749	[0.0502; 0.1374]	a
		A40	0.05827102	0.02097798	[0.0042; 0.1123]	a
		A80	0.10336237	0.0184999	[0.0557; 0.1510]	ab
	L5	C	0.24973916	0.01167623	[0.2197; 0.2798]	b
		J20	0.1778906	0.01557746	[0.1378; 0.2180]	a
		J40	0.25006196	0.01499042	[0.2114; 0.2887]	b
		A40	0.16089601	0.01641357	[0.1186; 0.2032]	a
		A80	0.25653723	0.01569823	[0.2161; 0.2970]	b

Table S15 Full statistical overview of the female Weibull survival model for the Starvation experiment, showing fixed effects, random effects, and sample size. Line 1, Generation F0 and the control treatment are used as the model baseline. Significant p-values are shown in bold.

Female Starvation Model					
Family: Weibull					
Distance ~ Treat * Gen + Treat * Line + Line * Gen + (1 | $Dish_{ID}$)					
Fixed effects	Estimate	Std. Error	Naïve Std. Error	z value	P-value
(Intercept)	4.04812	0.10025	0.04166	40.38	**< 0.0001**
TreatJ20	0.01127	0.1207	0.05279	0.09	0.9256
TreatJ40	-0.17254	0.11891	0.05926	-1.45	0.1468
TreatA40	0.00763	0.12293	0.05837	0.06	0.9505
TreatA80	-0.08971	0.11957	0.06384	-0.75	0.4531
GenF1	-0.26941	0.11747	0.05286	-2.29	**0.0218**
GenF2	0.05063	0.10583	0.04641	0.48	0.6324
LineL2	0.05699	0.11173	0.06042	0.51	0.6100
LineL3	0.22538	0.13823	0.05067	1.63	0.1030
LineL4	-0.20862	0.17817	0.05275	-1.17	0.2416
LineL5	-0.32494	0.11525	0.05112	-2.82	**0.0048**
TreatJ20:GenF1	0.25167	0.17681	0.05509	1.42	0.1546
TreatJ40:GenF1	0.27596	0.11805	0.0594	2.34	**0.0194**
TreatA40:GenF1	0.00792	0.11393	0.05103	0.07	0.9446
TreatA80:GenF1	0.00312	0.10099	0.05216	0.03	0.9754
TreatJ20:GenF2	-0.04221	0.12881	0.04929	-0.33	0.7431
TreatJ40:GenF2	-0.00654	0.11521	0.05529	-0.06	0.9547
TreatA40:GenF2	-0.10512	0.12528	0.05013	-0.84	0.4014
TreatA80:GenF2	0.05042	0.10794	0.05168	0.47	0.6404
TreatJ20:LineL2	0.0381	0.13685	0.06935	0.28	0.7807
TreatJ40:LineL2	0.10616	0.11614	0.07315	0.91	0.3607
TreatA40:LineL2	0.18525	0.106	0.07147	1.75	0.0805
TreatA80:LineL2	0.17487	0.11803	0.0723	1.48	0.1385
TreatJ20:LineL3	0.11017	0.16119	0.06285	0.68	0.4943
TreatJ40:LineL3	0.07707	0.14936	0.07267	0.52	0.6059
TreatA40:LineL3	0.11277	0.12998	0.06467	0.87	0.3856
TreatA80:LineL3	-0.01693	0.14432	0.06967	-0.12	0.9066
TreatJ20:LineL4	-0.3616	0.1895	0.06472	-1.91	0.0564
TreatJ40:LineL4	-0.0835	0.13668	0.06956	-0.61	0.5412
TreatA40:LineL4	0.0361	0.1409	0.06316	0.26	0.7978
TreatA80:LineL4	-0.10371	0.15433	0.07221	-0.67	0.5016
TreatJ20:LineL5	-0.27406	0.13889	0.06126	-1.97	**0.0485**
TreatJ40:LineL5	-0.04734	0.13502	0.06611	-0.35	0.7259
TreatA40:LineL5	0.06191	0.15186	0.06741	0.41	0.6835
TreatA80:LineL5	0.01588	0.11929	0.0667	0.13	0.8941
GenF1:LineL2	-0.3751	0.10603	0.0581	-3.54	**0.0004**
GenF2:LineL2	-0.36881	0.089	0.05612	-4.14	**< 0.0001**
GenF1:LineL3	-0.10141	0.12511	0.05786	-0.81	0.4176

Female Starvation Model					
Family: Weibull					
Distance ~ Treat * Gen + Treat * Line + Line * Gen + (1 | $Dish_{ID}$)					
Fixed effects	Estimate	Std. Error	Naïve Std. Error	z value	P-value
GenF2:LineL3	-0.22755	0.11884	0.05318	-1.91	0.0555
GenF1:LineL4	0.15788	0.16687	0.05997	0.95	0.3441
GenF2:LineL4	-0.05352	0.17158	0.05694	-0.31	0.7551
GenF1:LineL5	0.25025	0.13741	0.05803	1.82	0.0686
GenF2:LineL5	0.08711	0.10101	0.05126	0.86	0.3885
Log(scale)	-1.28493	0.04235	0.01769	-30.34	**< 0.0001**
Number of obs.: 1875, groups: $Dish_{ID}$, 128					

Table S16 Overview of means and confidence interval averaged over lines in the post hoc analysis of the female Weibull survival model for the Starvation experiment. Shows the mean, standard errors, 95%-confidence interval and groupings as determined by treatment contrasts.

Generation	Treatment	Mean	Std. Error	95% CI	Group
F0	C	54.48280468	3.861501575	[45.3827; 65.4076]	ab
	J20	49.98281856	4.408770902	[39.8148; 62.7475]	ab
	J40	46.33141261	2.728739876	[39.8035; 53.9299]	a
	A40	59.4256123	3.459445076	[51.14259; 69.0501]	b
	A80	50.5115019	1.853728258	[45.9508; 55.5248]	ab
F1	C	41.0504917	1.87412213	[36.4916; 46.1789]	ab
	J20	48.43703301	5.140561708	[36.8409; 63.6831]	ab
	J40	46.00272076	2.489986744	[40.0102; 52.8928]	b
	A40	45.1308386	1.991711883	[40.2766; 50.5701]	b
	A80	38.1771931	1.457810082	[34.5973; 42.1275]	a
F2	C	51.21144401	3.43009125	[43.0885; 60.8657]	a
	J20	45.03975784	1.697593172	[40.8685; 49.6368]	a
	J40	43.26539746	2.102091021	[38.1709; 49.0398]	a
	A40	50.2837949	2.089208678	[45.1753; 55.97]	a
	A80	49.93374291	2.062056778	[44.89; 55.5442]	a

Table S17 Overview of means and confidence interval in the post hoc analysis of the female Weibull survival model for the Starvation experiment. Shows the mean, standard errors, 95%-confidence interval and groupings as determined by treatment contrasts.

Generation	Line	Treatment	Mean	Std. Error	95% CI	Group
F0	L1	C	57.2898825	5.74312161	[25.468; 43.815]	a
		J20	57.9392695	4.64094475	[16.293; 43.213]	a
		J40	48.2107286	4.51922844	[20.766; 37.710]	a
		A40	57.7289034	6.30861541	[29.564; 43.168]	a
		A80	52.3744104	5.32899711	[22.585; 43.769]	a
	L2	C	60.6496561	5.41687619	[20.858; 29.849]	bc
		J20	63.7194189	6.40600991	[15.981; 23.440]	a
		J40	56.7543008	3.93871932	[15.377; 26.276]	ab
		A40	73.5523497	5.36467556	[30.715; 43.637]	d
		A80	66.041025	2.56260546	[21.693; 44.065]	cd
	L3	C	71.7724044	8.30224952	[25.533; 43.643]	ab
		J20	81.0395289	7.43247165	[21.617; 39.518]	ab
		J40	65.237122	7.19718675	[26.011; 38.461]	a
		A40	80.9562458	7.15863192	[34.326; 47.726]	b
		A80	64.5129396	5.7110805	[18.608; 54.117]	ab
	L4	C	46.5022448	7.72605921	[16.017; 22.898]	ab
		J20	32.7588661	7.73964532	[13.847; 19.594]	a
		J40	35.9976903	5.65174533	[13.841; 22.664]	ab
		A40	48.5811819	4.14204497	[20.225; 29.540]	c
		A80	38.32421	3.09454163	[17.278; 36.762]	bc
	L5	C	41.3961158	3.70091703	[25.717; 33.474]	b
		J20	31.8297423	4.20335147	[17.411; 27.057]	a
		J40	33.2250626	2.19722958	[19.938; 26.492]	a
		A40	44.3772137	5.80159444	[29.150; 38.671]	b
		A80	38.4501833	2.30527689	[21.229; 37.766]	ab
F1	L1	C	43.7598271	4.3737723	[39.488; 55.426]	a
		J20	56.9204814	9.44125731	[38.903; 55.285]	a
		J40	48.5277806	2.19256574	[43.871; 54.642]	a
		A40	44.4459102	2.26728552	[35.207; 50.454]	a
		A80	40.1302272	3.64657239	[33.258; 48.774]	a
	L2	C	31.8362710	2.445962	[23.319; 30.042]	a
		J20	43.0193199	7.31116326	[22.194; 29.579]	a
		J40	39.2592228	1.82108673	[20.216; 35.099]	ab
		A40	38.9162722	1.90786335	[28.124; 38.052]	b
		A80	34.7746255	1.93622413	[24.066; 37.392]	ab
	L3	C	49.5350851	3.77214965	[23.425; 39.469]	a
		J20	71.9366499	14.4018462	[24.858; 44.408]	a
		J40	59.3334061	5.06441806	[30.467; 42.521]	a
		A40	56.3179204	2.69005632	[26.382; 36.562]	a
		A80	44.6639424	2.71262883	[19.250; 36.313]	a
	L4	C	41.5948846	3.83146101	[18.201; 22.275]	a
		J20	37.6871026	3.38805022	[16.914; 27.622]	a

Generation	Line	Treatment	Mean	Std. Error	95% CI	Group
F1	L4	J40	42.4316295	2.90388564	[19.005; 28.481]	a
		A40	43.8000809	4.65147409	[18.590; 25.211]	a
		A80	34.3869789	2.79372743	[19.895; 29.531]	a
	L5	C	40.6107668	3.66632353	[23.051; 32.305]	a
		J20	40.1617475	3.61083068	[19.215; 33.030]	a
		J40	42.9532938	6.96452358	[18.526; 38.493]	a
		A40	43.8816004	5.76999765	[19.658; 35.200]	a
		A80	37.8385788	3.51093839	[18.192; 31.894]	a
F2	L1	C	60.2652751	4.20417846	[33.590; 42.492]	a
		J20	58.4292035	3.4889864	[29.068; 46.774]	a
		J40	50.383751	4.69879609	[32.226; 41.953]	a
		A40	54.6675153	3.28751156	[26.100; 36.422]	a
		A80	57.9434877	5.10760813	[30.404; 41.267]	a
	L2	C	44.1211213	3.77826484	[19.280; 26.132]	a
		J20	44.4383314	2.93074817	[17.056; 26.834]	a
		J40	41.0180081	4.25414229	[17.072; 25.851]	a
		A40	48.1683273	2.57787415	[22.654; 27.881]	a
		A80	50.5275542	2.46823675	[23.597; 32.531]	a
	L3	C	60.1343166	8.92114758	[20.044; 39.148]	a
		J20	65.0922871	5.36127293	[24.939; 36.417]	a
		J40	54.3021804	4.62943913	[25.618; 37.118]	a
		A40	61.0607396	4.82523434	[19.706; 34.091]	a
		A80	56.8470575	3.13046119	[18.393; 38.256]	a
	L4	C	46.3681798	6.0095624	[18.406; 24.014]	a
		J20	31.3143015	4.14774057	[18.371; 26.874]	a
		J40	35.659755	1.90153074	[19.332; 26.397]	a
		A40	43.6074831	2.66575858	[16.133; 25.990]	a
		A80	40.1897786	5.03800901	[19.486; 38.984]	a
	L5	C	47.50934	3.51552922	[21.971; 26.286]	a
		J20	35.0203325	3.25908868	[18.192; 26.228]	a
		J40	37.8828637	2.41725065	[18.152; 26.350]	a
		A40	45.848618	6.14574419	[17.169; 25.650]	a
		A80	46.4102696	2.47365806	[18.246; 29.250]	a

Table S18 Full statistical overview of the male lognormal survival model for the Starvation experiment, showing fixed effects, random effects, and sample size. Line 1, Generation F0 and the control treatment are used as the model baseline. Significant p-values are shown in bold.

Male Starvation Model					
Family: Lognormal					
Distance ~ Treat * Gen + Treat * Line + Line * Gen + (1 | $Dish_{ID}$)					
Fixed effects	Estimate	Std. Error	Naïve Std. Error	z value	P-value
(Intercept)	3.5087	0.105201	0.045359	33.35	**< 0.0001**
TreatJ20	-0.230279	0.147688	0.052321	-1.56	0.1189
TreatJ40	-0.177085	0.099578	0.056798	-1.78	0.0754
TreatA40	0.067139	0.08386	0.055336	0.8	0.4234
TreatA80	-0.060603	0.125748	0.059324	-0.48	0.6299
GenF1	0.336819	0.112302	0.047953	3	**0.0027**
GenF2	0.123076	0.111145	0.048061	1.11	0.2681
LineL2	-0.291737	0.11762	0.053193	-2.48	**0.0131**
LineL3	-0.000688	0.146578	0.05721	0	0.9963
LineL4	-0.556367	0.119062	0.052658	-4.67	**< 0.0001**
LineL5	-0.129729	0.109339	0.052797	-1.19	0.2354
TreatJ20:GenF1	0.221546	0.103961	0.045942	2.13	**0.0331**
TreatJ40:GenF1	0.222592	0.101126	0.049878	2.2	**0.0277**
TreatA40:GenF1	-0.171507	0.075483	0.047297	-2.27	**0.0231**
TreatA80:GenF1	-0.089171	0.125555	0.050996	-0.71	0.4776
TreatJ20:GenF2	0.20599	0.096749	0.044224	2.13	**0.0333**
TreatJ40:GenF2	0.149978	0.082257	0.049267	1.82	0.0683
TreatA40:GenF2	-0.270355	0.074343	0.051751	-3.64	**0.0003**
TreatA80:GenF2	-0.003845	0.135481	0.051421	-0.03	0.9774
TreatJ20:LineL2	-0.023757	0.124204	0.058708	-0.19	0.8483
TreatJ40:LineL2	-0.039113	0.107732	0.060054	-0.36	0.7166
TreatA40:LineL2	0.316227	0.086746	0.061692	3.65	**0.0003**
TreatA80:LineL2	0.274974	0.102494	0.063458	2.68	**0.0073**
TreatJ20:LineL3	0.097375	0.157901	0.059114	0.62	0.5375
TreatJ40:LineL3	0.123161	0.117128	0.061951	1.05	0.2930
TreatA40:LineL3	0.125544	0.123935	0.065726	1.01	0.3111
TreatA80:LineL3	0.009946	0.173671	0.065901	0.06	0.9543
TreatJ20:LineL4	0.079615	0.125256	0.055058	0.64	0.5250
TreatJ40:LineL4	0.098967	0.085739	0.060538	1.15	0.2484
TreatA40:LineL4	0.176844	0.093356	0.059868	1.89	0.0582
TreatA80:LineL4	0.335227	0.125053	0.0634	2.68	**0.0074**
TreatJ20:LineL5	-0.071182	0.129497	0.059451	-0.55	0.5825
TreatJ40:LineL5	-0.067139	0.10251	0.059306	-0.65	0.5125
TreatA40:LineL5	0.067675	0.098004	0.063402	0.69	0.4899
TreatA80:LineL5	0.025005	0.105032	0.063916	0.24	0.8118
GenF1:LineL2	-0.277849	0.124248	0.051071	-2.24	**0.0253**
GenF2:LineL2	-0.228912	0.126193	0.052013	-1.81	0.0697
GenF1:LineL3	-0.430172	0.137218	0.051881	-3.13	**0.0017**

Male Starvation Model					
Family: Lognormal					
Distance ~ Treat * Gen + Treat * Line + Line * Gen + (1 | $Dish_{ID}$)					
Fixed effects	Estimate	Std. Error	Naïve Std. Error	z value	P-value
GenF2:LineL3	-0.298445	0.15166	0.054913	-1.97	0.0491
GenF1:LineL4	-0.286661	0.125722	0.052145	-2.28	**0.0226**
GenF2:LineL4	-0.029756	0.129344	0.05397	-0.23	0.8181
GenF1:LineL5	-0.409332	0.134106	0.05443	-3.05	**0.0023**
GenF2:LineL5	-0.322676	0.117294	0.050728	-2.75	**0.0059**
Log(scale)	-1.376567	0.039459	0.017058	-34.89	**< 0.0001**
Number of obs.: 1758, groups: $Dish_{ID}$, 138					

Table S19 Overview of means and confidence interval averaged over lines in the post hoc analysis of the male lognormal survival model for the Starvation experiment. Shows the mean, standard errors, 95%-confidence interval and groupings as determined by treatment contrasts.

Generation	Treatment	Mean	Std. Error	95% CI	Group
F0	C	27.46728503	1.198797208	[24.5436; 30.7393]	a
	J20	22.17856478	1.61843991	[18.3743; 26.7705]	a
	J40	23.54905785	1.256964647	[20.5209; 27.0241]	a
	A40	33.69646517	0.943300952	[31.3497; 36.2189]	b
	A80	29.41259263	3.157956326	[22.2993; 38.7950]	ab
F1	C	29.04979549	0.998294187	[26.5863; 31.7416]	a
	J20	29.27365253	1.541185346	[25.5574; 33.5303]	a
	J40	31.115112	1.848505713	[26.6955; 36.2664]	a
	A40	30.02112901	1.290206477	[26.8718; 33.5395]	a
	A80	28.45340086	1.340211059	[25.1990; 32.1281]	a
F2	C	26.05250406	1.034932579	[23.5159; 28.8628]	a
	J20	25.84802139	1.105510074	[23.1488; 28.8619]	a
	J40	25.95028245	0.86043945	[23.8237; 28.2667]	a
	A40	24.38957553	1.148685068	[21.6003; 27.5391]	a
	A80	27.79055754	1.655488249	[23.8333; 32.4049]	a

Table S20 Overview of means and confidence interval in the post hoc analysis of the male lognormal survival model for the Starvation experiment. Shows the mean, standard errors, 95%-confidence interval and groupings as determined by treatment contrasts.

Generation	Line	Treatment	Mean	Std. Error	95% CI	Group
F0	L1	C	57.289882	5.743122	[44.240;74.189]	a
		J20	57.939269	4.640945	[47.128;71.231]	a
		J40	48.210729	4.519228	[37.859;61.393]	a
		A40	57.728903	6.308615	[43.553;76.519]	a
		A80	52.374410	5.328997	[40.288;68.086]	a
	L2	C	60.649656	5.416876	[48.174;76.356]	a
		J20	63.719419	6.406010	[49.169;82.576]	a
		J40	56.754301	3.938719	[47.455;67.876]	a
		A40	73.552350	5.364676	[60.942;88.772]	a
		A80	66.041025	2.562605	[59.753;72.991]	a
	L3	C	71.772404	8.302250	[53.262;96.715]	a
		J20	81.039529	7.432472	[63.972; 102.66]	a
		J40	65.237122	7.197187	[49.085;86.704]	a
		A40	80.956246	7.158632	[64.451;101.69]	a
		A80	64.512940	5.711081	[51.347;81.055]	a
	L4	C	46.502245	7.726059	[30.298;71.372]	a
		J20	32.758866	7.739645	[17.814;60.242]	a
		J40	35.997690	5.651745	[24.014;53.963]	a
		A40	48.581182	4.142045	[38.993;60.527]	a
		A80	38.324210	3.094542	[31.121;47.195]	a
	L5	C	41.396116	3.700917	[32.873;52.129]	a
		J20	31.829742	4.203351	[22.644;44.742]	a
		J40	33.225063	2.197230	[28.016;39.402]	a
		A40	44.377214	5.801594	[31.678;62.167]	a
		A80	38.450183	2.305277	[32.943;44.879]	a
F1	L1	C	43.759827	4.373772	[33.818;56.624]	a
		J20	56.920481	9.441257	[37.113;87.3]	a
		J40	48.527781	2.192566	[43.191;54.524]	a
		A40	44.445910	2.267286	[38.968;50.694]	a
		A80	40.130227	3.646572	[31.748;50.726]	a
	L2	C	31.836271	2.445962	[26.115;38.811]	a
		J20	43.019320	7.311163	[27.755;66.678]	a
		J40	39.259223	1.821087	[34.834;44.247]	a
		A40	38.916272	1.907863	[34.295;44.16]	a
		A80	34.774625	1.936224	[30.124;40.143]	a
	L3	C	49.535085	3.772150	[40.704;60.282]	ab
		J20	71.936650	14.401846	[42.93;120.54]	ab
		J40	59.333406	5.064418	[47.612;73.941]	b
		A40	56.317920	2.690056	[49.792;63.7]	b
		A80	44.663942	2.712629	[38.19;52.236]	a
	L4	C	41.594885	3.831461	[32.801;52.746]	a
		J20	37.687103	3.388050	[29.89;47.519]	a

Generation	Line	Treatment	Mean	Std. Error	95% CI	Group
F1	L4	J40	42.431630	2.903886	[35.567;50.621]	a
		A40	43.800081	4.651474	[33.308;57.597]	a
		A80	34.386979	2.793727	[27.888;42.401]	a
	L5	C	40.610767	3.666324	[32.177;51.255]	a
		J20	40.161748	3.610831	[31.852;50.64]	a
		J40	42.953294	6.964524	[28.276;65.248]	a
		A40	43.881600	5.769998	[31.263;61.593]	a
		A80	37.838579	3.510938	[29.787;48.066]	a
F2	L1	C	60.265275	4.204178	[50.344;72.142]	a
		J20	58.429203	3.488986	[50.091;68.155]	a
		J40	50.383751	4.698796	[39.615;64.081]	a
		A40	54.667515	3.287512	[46.815;63.837]	a
		A80	57.943488	5.107608	[46.163;72.73]	a
	L2	C	44.121121	3.778265	[35.379;55.023]	a
		J20	44.438331	2.930748	[37.489;52.676]	a
		J40	41.018008	4.254142	[31.393;53.594]	a
		A40	48.168327	2.577874	[41.96;55.296]	a
		A80	50.527554	2.468237	[44.548;57.31]	a
	L3	C	60.134317	8.921148	[41.02;88.157]	a
		J20	65.092287	5.361273	[52.638;80.494]	a
		J40	54.302180	4.629439	[43.586;67.653]	a
		A40	61.060740	4.825234	[49.805;74.861]	a
		A80	56.847057	3.130461	[49.322;65.52]	a
	L4	C	46.368180	6.009562	[33.196;64.767]	a
		J20	31.314302	4.147741	[22.254;44.063]	a
		J40	35.659755	1.901531	[31.079;40.916]	a
		A40	43.607483	2.665759	[37.248;51.053]	a
		A80	40.189779	5.038009	[29.09;55.526]	a
	L5	C	47.509340	3.515529	[39.257;57.497]	a
		J20	35.020332	3.259089	[27.549;44.518]	a
		J40	37.882864	2.417251	[32.136;44.658]	a
		A40	45.848618	6.145744	[32.45;64.779]	a
		A80	46.410270	2.473658	[40.451;53.248]	a

Figure S1 Per generation comparison between treatments for the activity model in Line 1, split between the sexes. Error bars show the 95% confidence interval, while the letters annotate significant groupings of the treatments, assigning similarity when treatments share a letter. Sidak p-adjustment was used for the post-hoc comparison.

Figure S2 Per generation comparison between treatments for the activity model in Line 2, split between the sexes. Error bars show the 95% confidence interval, while the letters annotate significant groupings of the treatments, assigning similarity when treatments share a letter. Sidak p-adjustment was used for the post-hoc comparison.

Figure S3 Per generation comparison between treatments for the activity model in Line 3, split between the sexes. Error bars show the 95% confidence interval, while the letters annotate significant groupings of the treatments, assigning similarity when treatments share a letter. Sidak p-adjustment was used for the post-hoc comparison.

Figure S4 Per generation comparison between treatments for the activity model in Line 4, split between the sexes. Error bars show the 95% confidence interval, while the letters annotate significant groupings of the treatments, assigning similarity when treatments share a letter. Sidak p-adjustment was used for the post-hoc comparison.

Figure S5 Per generation comparison between treatments for the activity model in Line 5, split between the sexes. Error bars show the 95% confidence interval, while the letters annotate significant groupings of the treatments, assigning similarity when treatments share a letter. Sidak p-adjustment was used for the post-hoc comparison.

Figure S6 Per generation comparison between treatments for the distance model in Line 1, split between the sexes. Error bars show the 95% confidence interval, while the letters annotate significant groupings of the treatments, assigning similarity when treatments share a letter. Sidak p-adjustment was used for the post-hoc comparison.

Figure S7 Per generation comparison between treatments for the distance model in Line 2, split between the sexes. Error bars show the 95% confidence interval, while the letters annotate significant groupings of the treatments, assigning similarity when treatments share a letter. Sidak p-adjustment was used for the post-hoc comparison.

Figure S8 Per generation comparison between treatments for the distance model in Line 3, split between the sexes. Error bars show the 95% confidence interval, while the letters annotate significant groupings of the treatments, assigning similarity when treatments share a letter. Sidak p-adjustment was used for the post-hoc comparison.

Figure S9 Per generation comparison between treatments for the distance model in Line 4, split between the sexes. Error bars show the 95% confidence interval, while the letters annotate significant groupings of the treatments, assigning similarity when treatments share a letter. Sidak p-adjustment was used for the post-hoc comparison.

Figure S10 Per generation comparison between treatments for the distance model in Line 5, split between the sexes. Error bars show the 95% confidence interval, while the letters annotate significant groupings of the treatments, assigning similarity when treatments share a letter. Sidak p-adjustment was used for the post-hoc comparison.

Treatment comparisons within Line L1 (Activity)

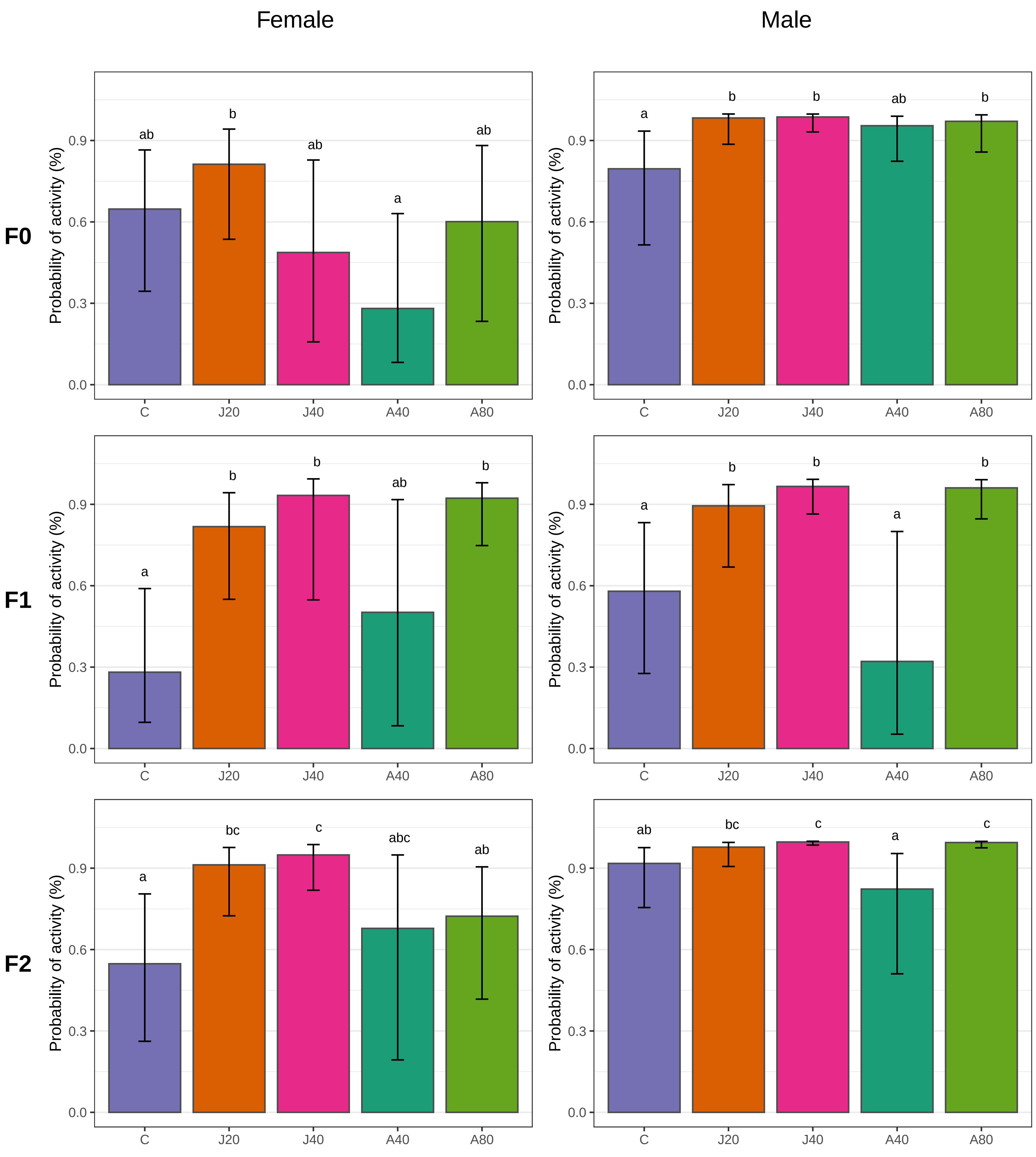

Treatment comparisons within Line L2 (Activity)

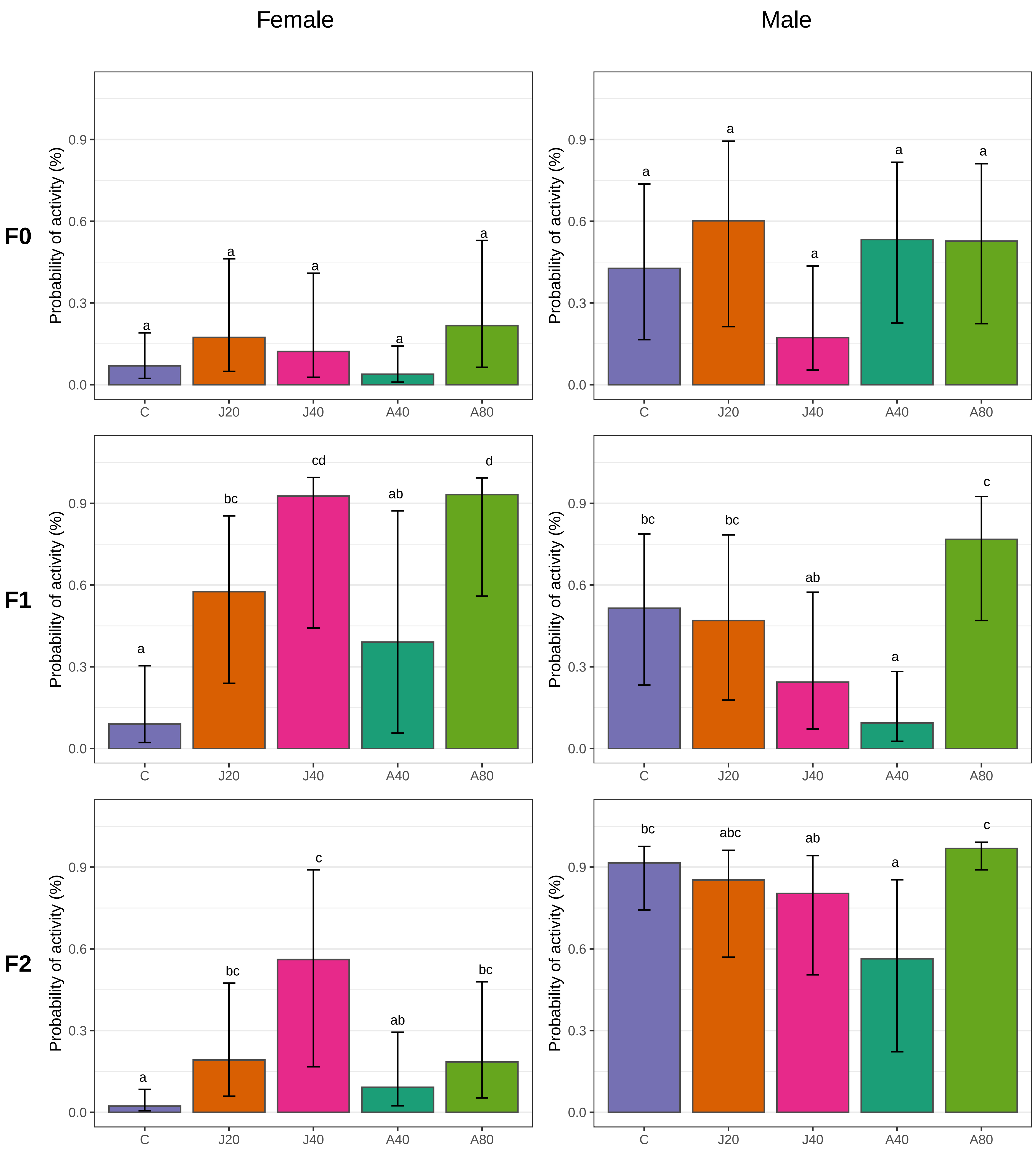

Treatment comparisons within Line L3 (Activity)

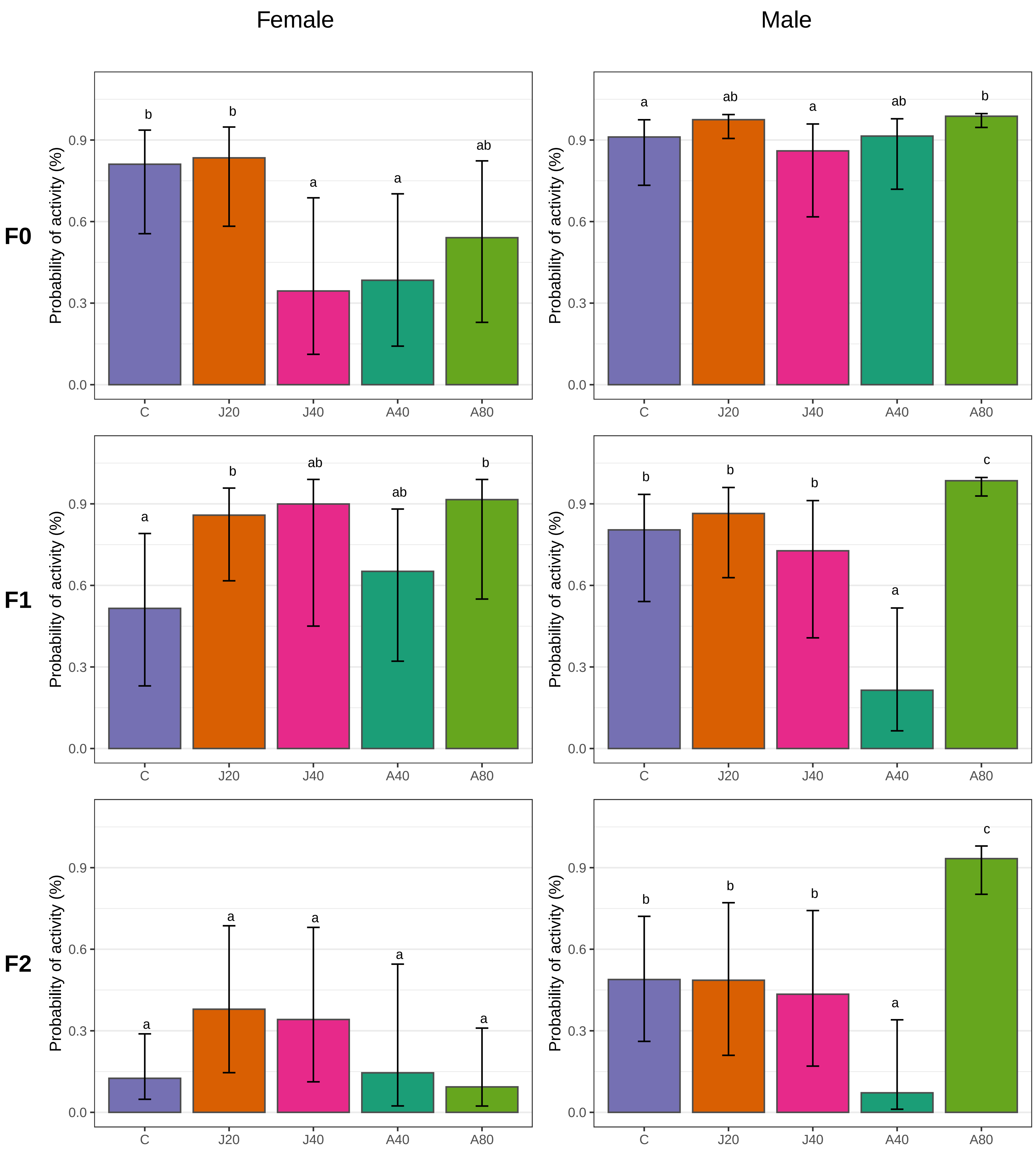

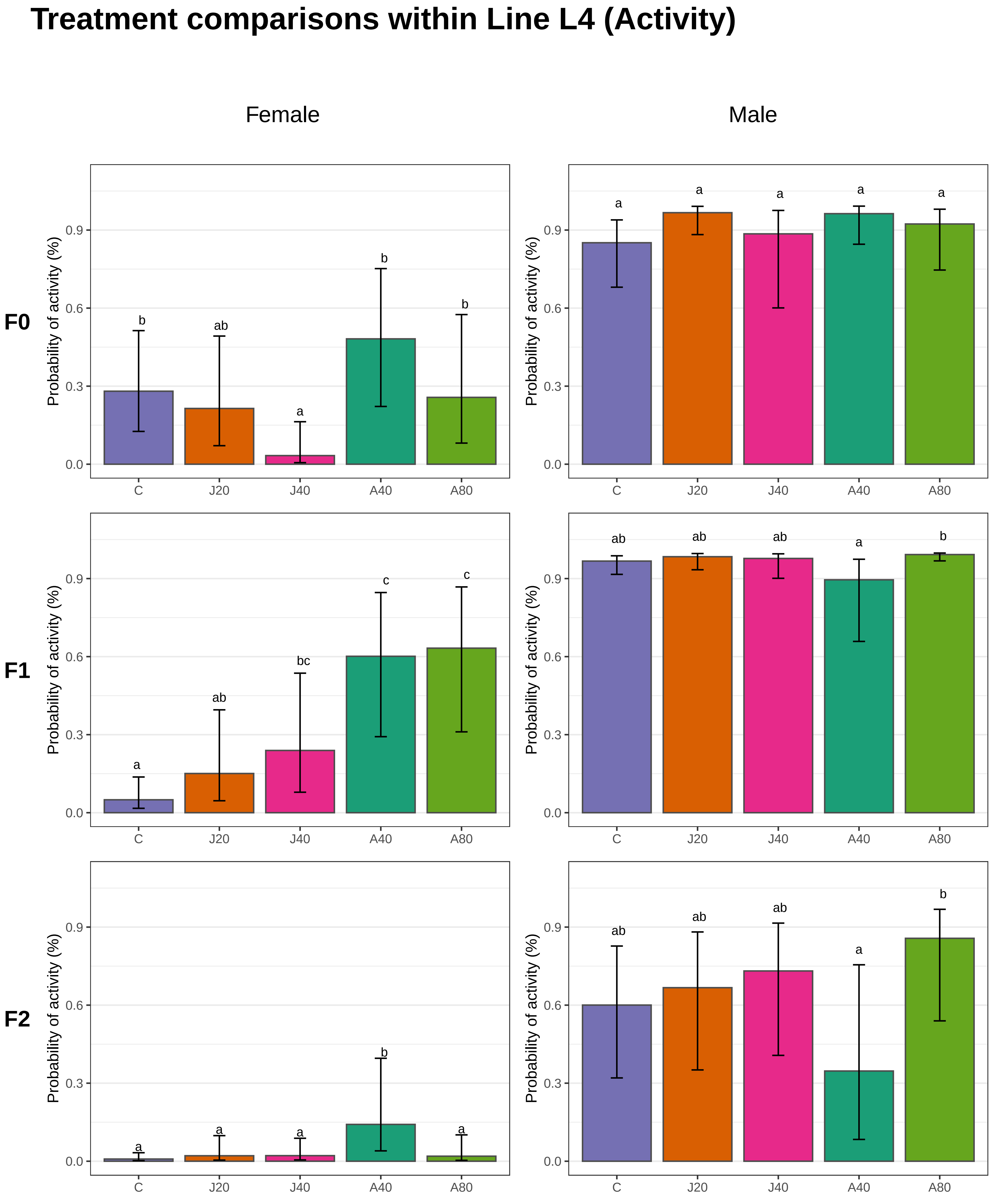
Treatment comparisons within Line L4 (Activity)
Female
Male
F0
F1
F2
Probability of activity (%)
0.0
0.3
0.6
0.9
C
J20
J40
A40
A80
b
ab
a
b
b
a
a
a
a
a
a
ab
bc
c
c
ab
ab
ab
a
b
a
a
a
b
a
ab
ab
ab
a
b

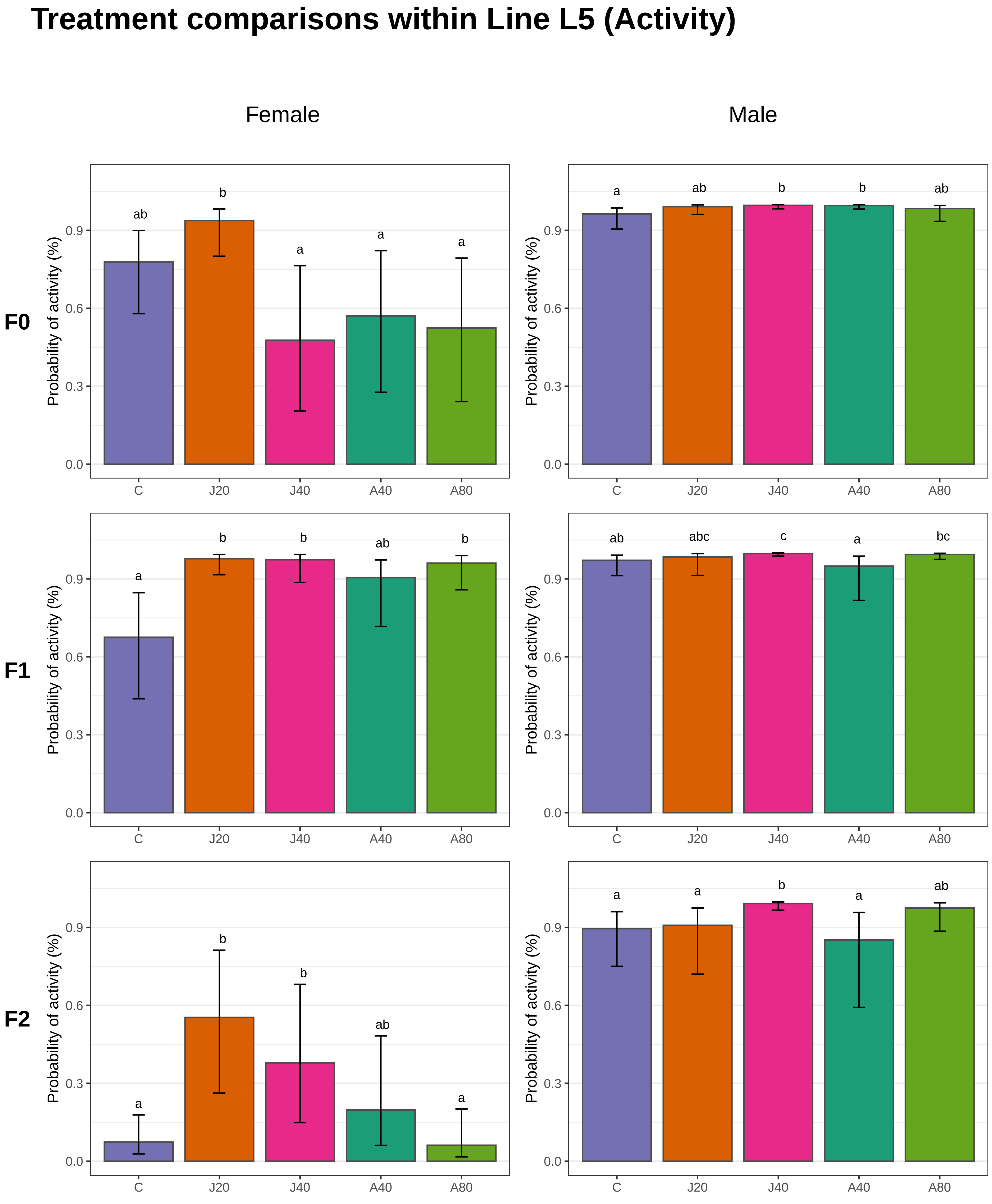
Treatment comparisons within Line L5 (Activity)
Female
Male
F0
F1
F2
Probability of activity (%)
0.0
0.3
0.6
0.9
C
J20
J40
A40
A80
ab
b
a
a
a
a
ab
b
b
ab
a
b
b
ab
b
ab
abc
c
a
bc
a
b
b
ab
a
a
a
b
a
ab

Treatment comparisons within Line L1 (Distance)

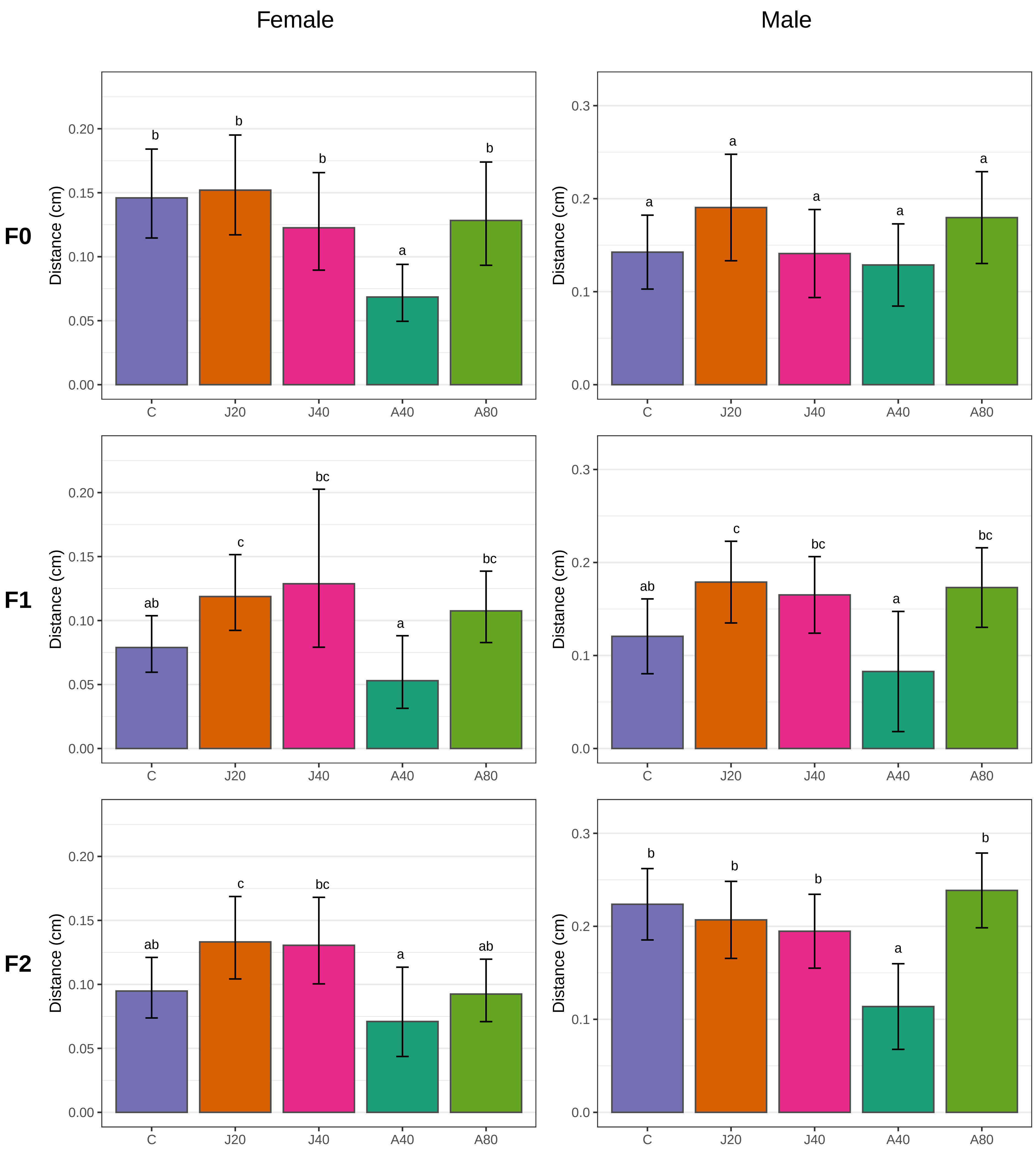

Treatment comparisons within Line L2 (Distance)

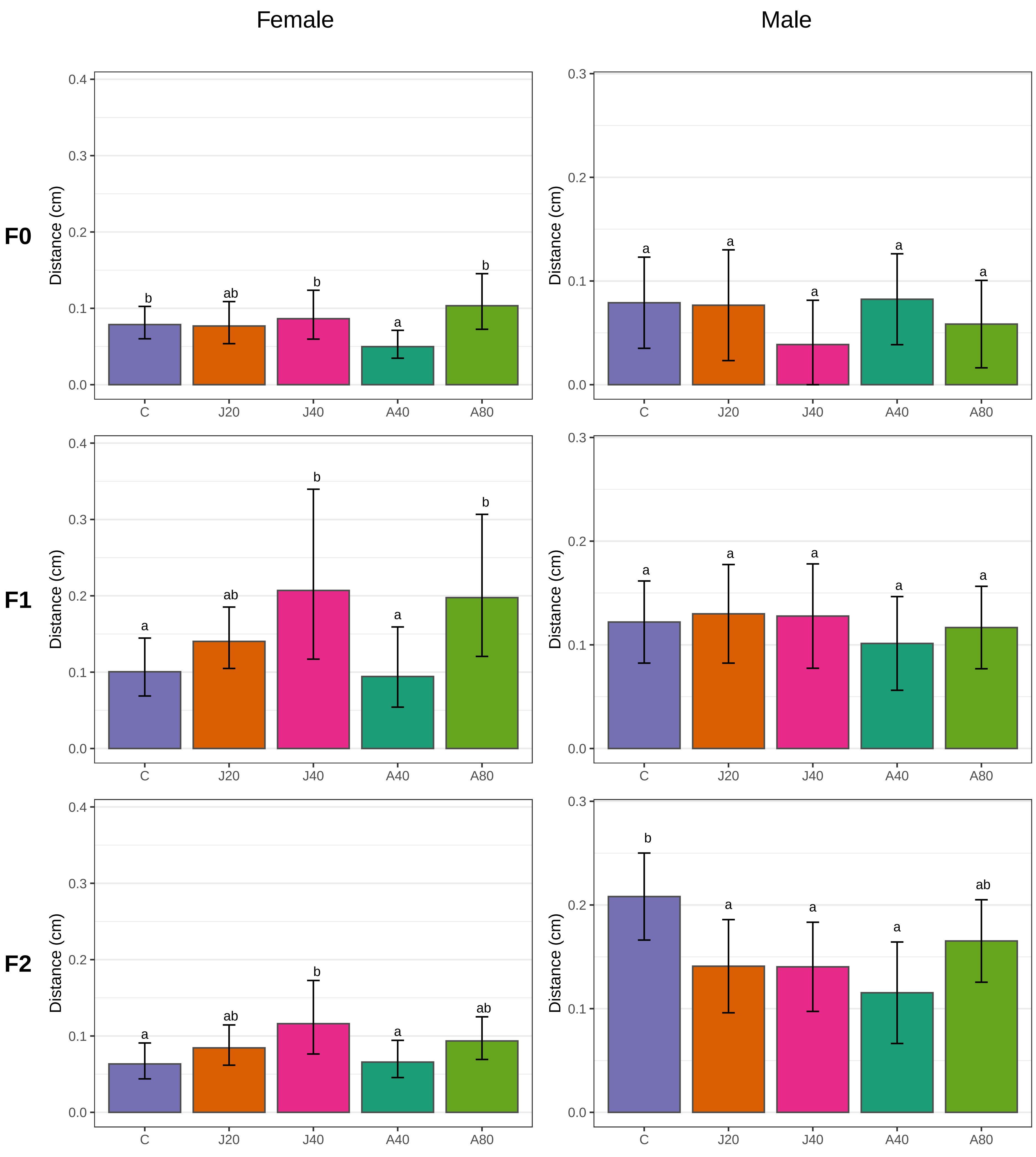

Treatment comparisons within Line L3 (Distance)

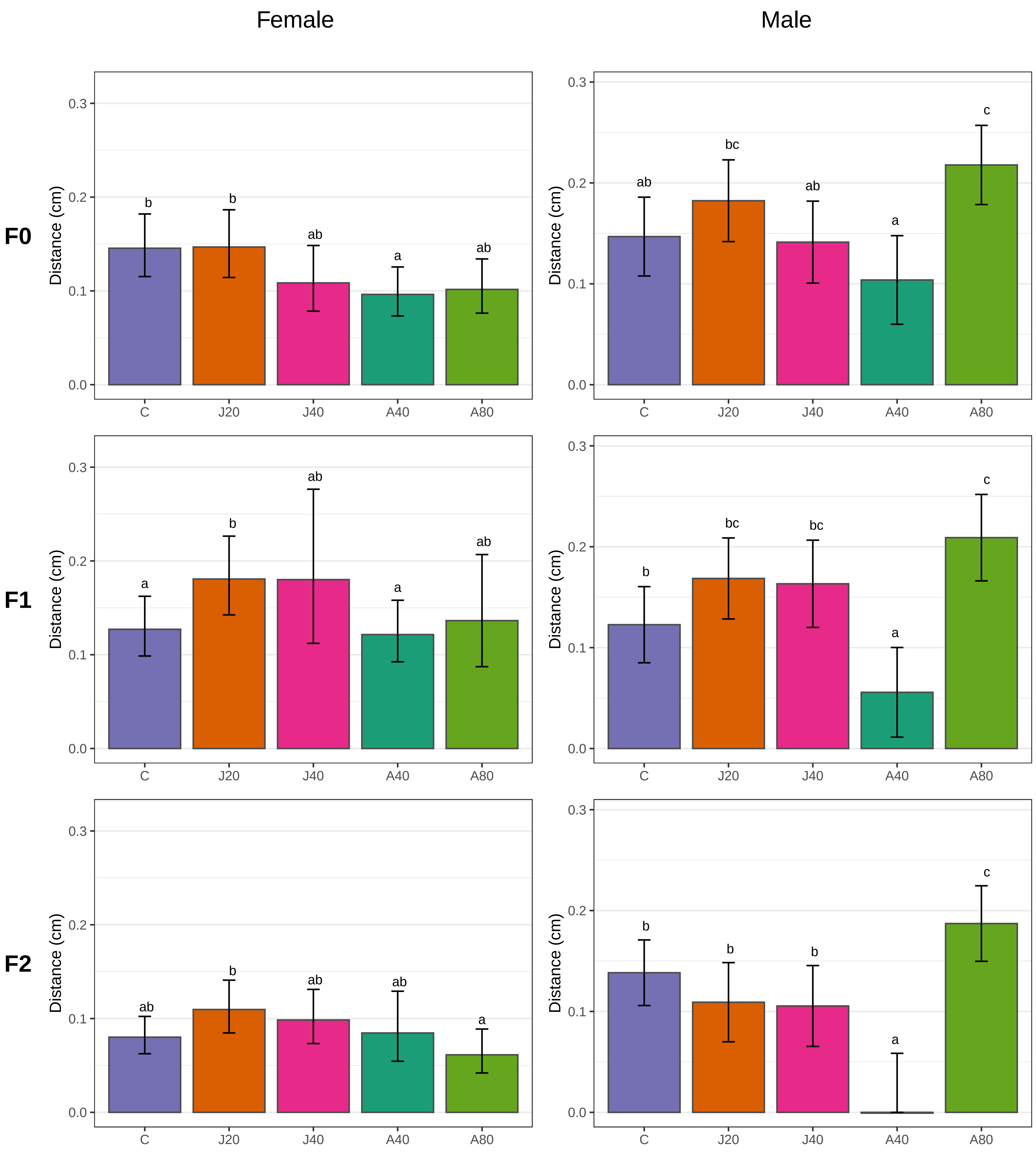

Treatment comparisons within Line L4 (Distance)

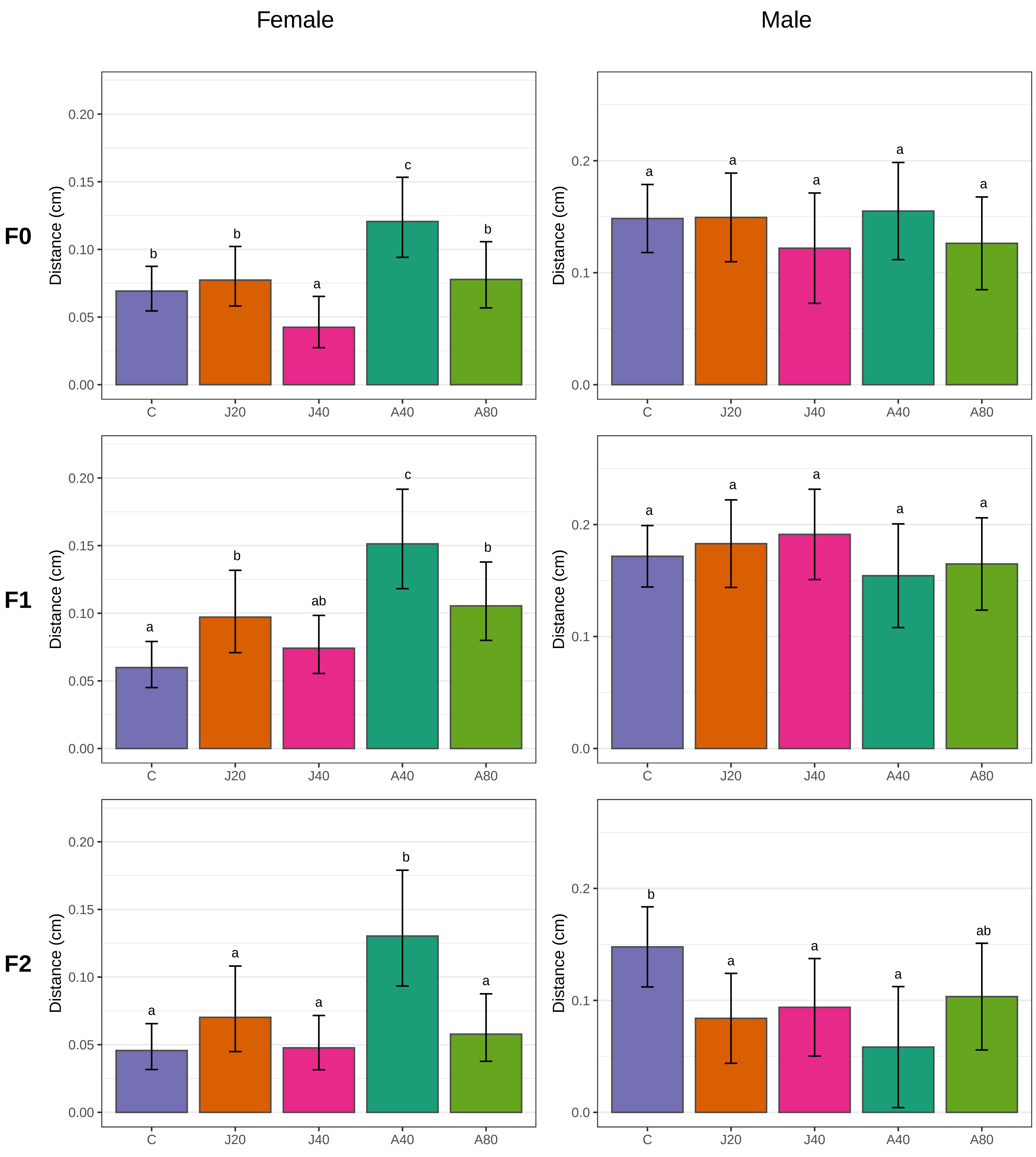

Treatment comparisons within Line L5 (Distance)

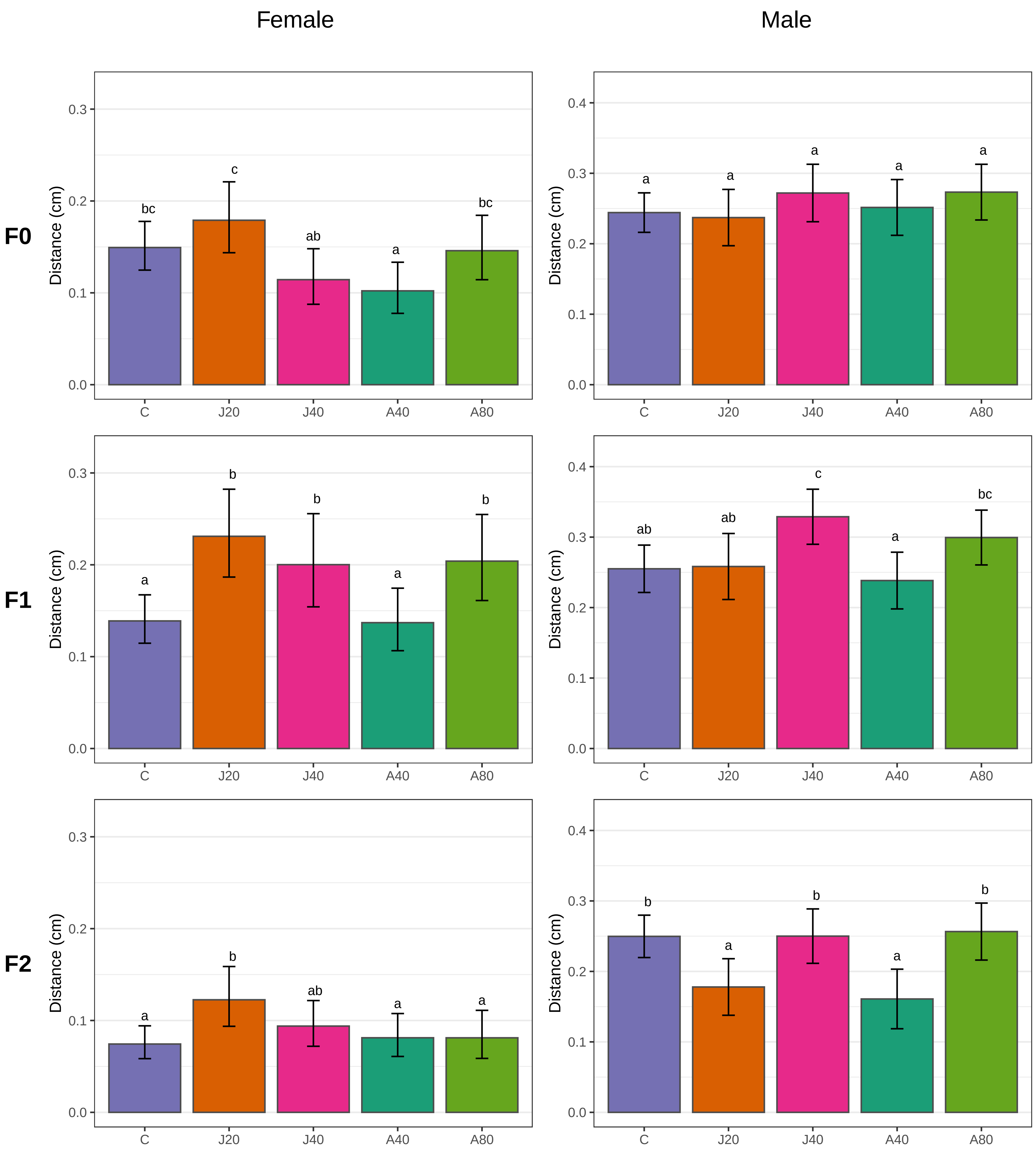

Supplement: Supplementary file 1 [file medicines-13-00024-s001.zip › medicines-4163825-supplementary.pdf]
